# Spontaneous non-canonical assembly of CcmK hexameric components from β-carboxysome shells of cyanobacteria

**DOI:** 10.1371/journal.pone.0185109

**Published:** 2017-09-21

**Authors:** Luis F. Garcia-Alles, Eric Lesniewska, Katharina Root, Nathalie Aubry, Nicolas Pocholle, Carlos I. Mendoza, Eric Bourillot, Konstantin Barylyuk, Denis Pompon, Renato Zenobi, David Reguera, Gilles Truan

**Affiliations:** 1 LISBP, CNRS, INRA, INSA, University of Toulouse, Toulouse, France; 2 ICB UMR CNRS 6303, University of Bourgogne Franche-Comte, Dijon, France; 3 Department of Chemistry and Applied Biosciences, ETH Zurich, Zurich, Switzerland; 4 Departament de Física de la Matèria Condensada, Universitat de Barcelona, Barcelona, Spain; 5 Instituto de Investigaciones en Materiales, Universidad Nacional Autónoma de México, Cd Mx, México; Martin-Luther-Universitat Halle-Wittenberg, GERMANY

## Abstract

CcmK proteins are major constituents of icosahedral shells of β-carboxysomes, a bacterial microcompartment that plays a key role for CO_2_ fixation in nature. Supported by the characterization of bidimensional (2D) layers of packed CcmK hexamers in crystal and electron microscopy structures, CcmK are assumed to be the major components of icosahedral flat facets. Here, we reassessed the validity of this model by studying CcmK isoforms from *Synechocystis sp*. *PCC6803*. Native mass spectrometry studies confirmed that CcmK are hexamers in solution. Interestingly, potential pre-assembled intermediates were also detected with CcmK2. Atomic-force microscopy (AFM) imaging under quasi-physiological conditions confirmed the formation of canonical flat sheets with CcmK4. Conversely, CcmK2 formed both canonical and striped-patterned patches, while CcmK1 assembled into remarkable supra-hexameric curved honeycomb-like mosaics. Mutational studies ascribed the propensity of CcmK1 to form round assemblies to a combination of two features shared by at least one CcmK isoform in most β-cyanobacteria: a displacement of an α helical portion towards the hexamer edge, where a potential phosphate binding funnel forms between packed hexamers, and the presence of a short C-terminal extension in CcmK1. All-atom molecular dynamics supported a contribution of phosphate molecules sandwiched between hexamers to bend CcmK1 assemblies. Formation of supra-hexameric curved structures could be reproduced in coarse-grained simulations, provided that adhesion forces to the support were weak. Apart from uncovering unprecedented CcmK self-assembly features, our data suggest the possibility that transitions between curved and flat assemblies, following cargo maturation, could be important for the biogenesis of β-carboxysomes, possibly also of other BMC.

## Introduction

Bacterial microcompartments (BMC) are proteinaceous organelles that sequester enzymes that catalyze processes involving toxic or volatile intermediates in a wide variety of bacteria [1]. Diverse metabolic pathways rely on BMC, among which are those sustaining the fixation of CO_2_ by photosynthetic cyanobacteria (Carboxysomes, CB), or the utilization of ethanolamine (Eut) or 1,2-propanediol (Pdu)[2]. CB is a BMC subtype that participates in a carbon-concentrating mechanism (Ccm) that allows photosynthetic cyanobacteria and chemoautotrophs to enhance carbon fixation yields [3]. For that, CB confine within their shells two key enzymatic activities: ribulose-1,5-bisphosphate (RuBP) carboxylase/oxygenase (RuBisCO) and carbonic anhydrases (CA). CB are classified in subtypes α and β, depending on whether RuBisCO Form 1A or 1B is encapsulated. Electron microscopy (EM) studies showed that CB are basically icosahedra, although size (70–600 nm), shape and composition variability were noticed, especially when imaging compartments within cells [4–8]. Apart from shell and mentioned enzymatic contents, CcmM and CcmN proteins of β-cyanobacteria provide scaffolding functions permitting to organize together cargo and shell of their β*-*CB. In a subset of these organisms, CcmM also contributes CA activity [7, 9]. Short peptide sequences of scaffolding/cargo proteins are known to mediate contacts with the shell [10, 11], something that allowed to engineer new BMC with reprogrammed contents [10, 12–14]. In terms of biogenesis, two fluorescence microscopy studies concurred to prove for β-CB that shell components assemble around preformed procarboxysome seeds composed of condensed RuBisCO complexes [15, 16]. Other studies, however, evidenced that BMC shell formation might be uncoupled from cargo content[14, 17, 18], a possibility that should not be fully ruled out for β-CB[19].

Compared to viral capsids, which can be built from a single or few subunits that adapt to “quasi-equivalent” environments of icosahedral capsids [20], BMC shell composition is more complex. Genomic data mining highlighted the presence of variable numbers of shell paralogs in every BMC-producing species, with a minimum of four components in the less complex cases[1]. Assembly bricks were grouped in three major classes [21, 22]: (i) pentamers (BMC-P, structural fold Pfam03319); (ii) hexamers (BMC-H); and (iii) pseudohexameric trimers (BMC-T), the last two deriving from the association of six single- or three bi-domain units, respectively, each domain displaying the hallmark BMC fold (Pfam00936). Abundant crystal structures proved the propensity of BMC-H homohexamers to assemble side-by-side into tightly packed 2D sheets [21, 22], something confirmed by EM data [23]. Accordingly, a structural model was proposed with BMC-H being major building blocks of flat icosahedral triangular facets and BMC-P occupying vertices. Other components, tentatively BMC-T, might compose edges between facets and/or conical joints to BMC-P vertices [24, 25]. Globally, this structural model was validated with the impressive recent elucidation of the first high-resolution crystal structure of *Haliangium ochraceum* (*HO*) BMC shells [26], which among other important observations, revealed the existence of four types of interfaces in the shells and the relative orientation of shell components. Cryo-electron tomography and AFM investigations on full CBs also indicated that shells have a thickness of 30 to 45 Å [4, 5, 27], which approximately corresponds to the width of a single layer of shell proteins.

Interactions established between assembling units of BMC shells are expected to be weak, similarly to what is measured for big protein assemblies like viral capsids [28]. Lateral contacts between hexamers revealed in 3D structures of BMC-H/T bury small areas, as compared to water-excluded surfaces in real dimers [29]. Attraction energies of about 2 *k*_*B*_*T* were calculated between CcmK2 hexamers in atomistic simulations[30], and comparable energetic regimes permitted to reproduce BMC assembly in coarse-grained simulations [31]. Plasticity is observed when comparing arrangements within crystals. For example, up to 13 Ǻ subunit lateral displacements were revealed for homologous BMC-H/T proteins [32, 33], and variable packings were noticed between stacked layers characterized within a crystal structure [24] or different crystal forms of the same protein [33]^,^ [34]. Similarly, assemblies with hexamers forming strips in alternating orientations were characterized for CcmK4 *Syn6803* [35], contrasting with the “canonical” arrangement observed with CcmK4 *Syn7942* [36]. In line with this argumentation, two distinct geometries between interacting BMC-H hexamers, one flat, the other bent by about 30°, were found to occur in *HO* BMC shells [26]. Assembly plasticity might also be a feature of proteins that belong to Pfam03319. CcmL and CsoS4A crystallized as pentamers [37], while hexamers were characterized within crystals of EutN from *E*. *coli*, despite tertiary structure agreeing with the expected Pfam03319 fold [37, 38]. Such plasticity might be an important feature contributing to the measured considerably higher flexibility of β-carboxysomes, as compared to viruses or encapsulins [27].

Structural adaptability imposes additional caution in interpreting structural data. Molecular stress promoted by sample conditioning for EM studies or constraints imposed within crystals might lead to incomplete views of the system under study. Comparatively, atomic-force microscopy (AFM) is probably a less invasive approach. Proteins can be imaged in solution under quasi-physiological conditions. Interactions between bio-molecules and inorganic supports like mica are relatively weak and cantilever scanning forces can be tuned to minimize artifacts. Continuous monitoring is also feasible, something that gives access to the dynamics of the process under study, as indeed demonstrated recently by the high-speed (HS-) AFM characterization of the assembly dynamics of the single *HO* BMC-H protein [39]. In the present work, we followed an AFM approach to investigate the assembly of the hexameric CcmK isoforms from the model cyanobacteria, *Synechococcus sp*. *PCC6803* (called *Syn6803* hereafter). We opted to use oligohistidine tagged proteins. Despite seeming counterintuitive, since absence of tags is normally preferred, our choice pursued two potential advantages: i) the presence of cationic tags could mediate adsorption to the weak anionic support [40], opening the way for later investigations on combinations of proteins with variable isoelectric points, which in the absence of tags would adhere with variable strengths; ii) by distancing the assembly plane from the support, conformational freedom should increase and potential artifacts caused by direct contacts with the support might be limited. This strategy permitted the characterization of the assembly of *Syn6803* CcmK isoforms under similar conditions and revealed an unprecedented behavior for one of the dominant isoforms, CcmK1, which was found to organize as curved polygonal patches. Canonical flat sheets were characterized for CcmK2 and CcmK4, albeit the former also resulted in striped patches. Sequence comparisons followed by mutational work provided evidence to support the notion that two combined structural elements of CcmK1 are responsible for its unusual properties. Moreover, theoretical simulations sustained the weak preference of carboxysomal BMC-H proteins to form flat assemblies, and enabled us to reproduce the formation of curved assemblies. Overall, we provide evidence suggesting that BMC-H components could introduce curvature in BMC shells, something that could be of significance for BMC biogenesis/assembly.

## Results

### Design and preparation of CcmK constructs

Bearing in mind that purification tags could impact solubility and/or assembly behavior, short tetra-histidine (His_4_) peptides were placed at either N- or C-terminal sides of all four CcmK isoforms from *Syn6803*. A tobacco etch virus (TEV) protease recognition site was included between protein sequence and tags to facilitate the preparation of untagged versions, when necessary (S1 Fig). We will refer to such constructs as ^HT-^K and K^-TH^ to indicate tagging emplacement at either the N- or the C-terminus, respectively, and ^-^K or K^-^ to refer to the corresponding TEV-proteolysis products. Expression of the different constructs in *E coli* and solubility were monitored by SDS-PAGE (S2A and S2B Fig) and compared with untagged proteins (^Un^K, S2C Fig), which only differed from wild-type sequence by the presence of Ala replacing the second residue and by an extra Ala at the C-terminus. The four isoforms were well expressed, with two exceptions (^HT-^K1 and K4^-TH^) for which bands were fainter. After cellular lysis, CcmK2 and CcmK4 remained soluble irrespective of the presence and position of tags. Multiple bands were apparent for the K4^-TH^ construct, suggesting sensitivity to cellular proteases, something confirmed by the presence of multiple peaks detected by mass spectrometry (MS) on purified samples (see below). In the case of CcmK1, N-ter tags rendered the protein insoluble. In contrast to the other three isoforms, none of the tested constructs and conditions permitted expression of a soluble form of CcmK3. This was likely related to folding/oligomerization defects, since untagged ^Un^K3 also expressed well but remained fully insoluble after cell lysis (S2C Fig). This result is in agreement with the lack of reports on *in vitro* studies for this isoform.

The five soluble proteins, namely K1^-TH^, ^HT-^K2, K2^-TH^, ^HT-^K4 and K4^-TH^, were purified by immobilized metal ion affinity chromatography (IMAC) and used for subsequent studies.

### Characterization of CcmK isoforms in solution

The oligomerization behavior of purified constructs was first verified by size-exclusion chromatography (SEC). CcmK1 and CcmK4 isoforms eluted at volumes expected for hexamers, with molecular masses (MW) of 65 kDa for K1^-TH^, 72 kDa for ^HT-^K4 and 65kDa for K4^-TH^, as estimated by comparison with calibrated protein standards. On the other hand, K2^-TH^ elution volumes matched to a 100–110 kDa protein, suggesting equilibrium between hexamer and dodecamer species in solution, in agreement with previous reports [35, 41].

**Fig 1:**
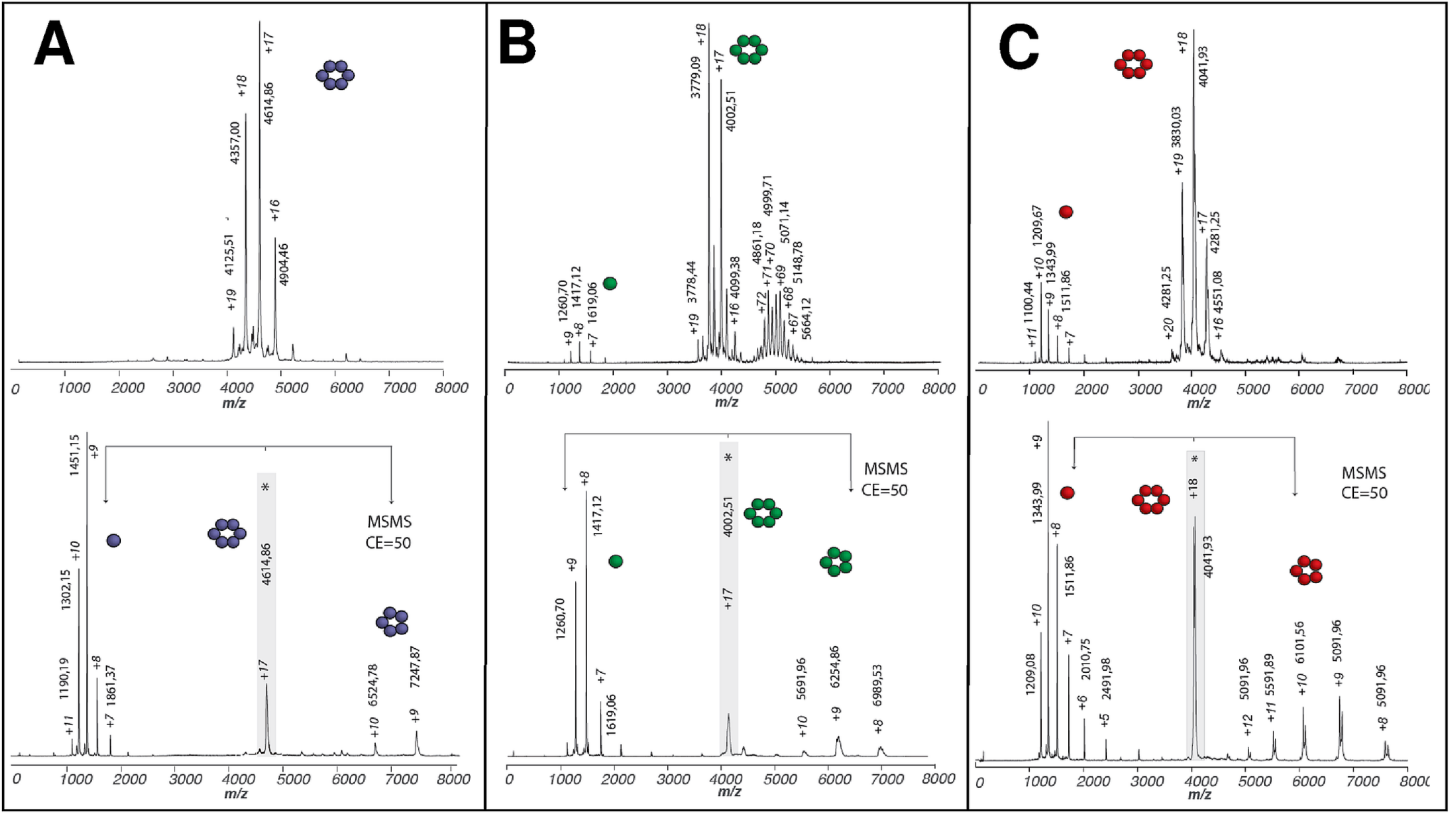
Oligomerization of CcmK1-4 and potential assembling intermediates with CcmK2 of *Syn6803*. Positive-ion mode native ESI-MS spectra from CcmK isoforms are presented on top panels: *A*, *Syn6803* K1^–^; *B*, *Syn6803* –K2*; C*, *Syn6803* –K4. The spectra are dominated by signals from multiply charged ions of CcmK hexamers. In addition, potential assembling intermediates with higher oligomerization state were noticed in experiments with−K2 (*B*). With all proteins, tags were removed by TEV protease treatments prior to spraying. Bottom panels present collisional activation data collected on hexamer precursor ions selected from top spectra (indicated with an asterisk). An asymmetric charge partitioning is noticed, hexamers dissociation resulting in monomer and pentamer species. Species m/z values and charges are indicated for major peaks. Cartoons schematically illustrate the stoichiometries of detected protein complexes and subcomplexes. Molecular weights of neutral species estimated from data measured for species with different charges are compiled in S1 Table.

Monomer association was next investigated by electrospray ionization native-MS (n-MS), a suitable approach for the characterization in solution of protein-ligand(-protein) interactions and the investigation of complex assembly phenomena, such as viral capsid formation [42]. Clusters of peaks in the 3500–5000 m/z range were detected as most predominant under soft ionization conditions for all CcmK proteins, which were pretreated with TEV protease to increase sample homogeneity (Fig 1 and S3 Fig). These signals corresponded to multiply charged ions whose mass is consistent with CcmK hexamers (S1 Table). These species were slightly heavier than masses calculated considering monomer data measured under more stringent conditions or from MS/MS spectra (see below). These deviations are caused by water molecules and salts that remain bound to proteins under soft ion-desolvation conditions required to preserve protein complexes. Tandem MS/MS experiments further confirmed the stoichiometry of the detected CcmK complexes. Discrete peaks with a single charge state were selected (indicated with asterisk in Fig 1 and S3 Fig) and subjected to collision-induced dissociation (CID). A common dissociation pattern was observed for all CcmK. Namely, peaks corresponding to monomers and pentamers appeared in the *m/z* region below or above, respectively, the parent ion peak. Sharper CID product ion peaks allowed us to attain more accurate mass measurements, as a consequence of dissociation of the residual solvent and salt adducts. Measured masses for monomers matched values calculated from amino acid sequences (S1 Table). Values with K1^-^, K2^-^ and K4^-^ indicated loss of the first Met residue (131.04 Da).

Remarkably, n-MS data for the two CcmK2 constructs revealed the presence of a cluster of peaks in the 4700–6300 m/z range. The MW of these species was estimated to range between 239–275 and 345–367 kDa for K2^-^ and ^-^K2, respectively. These values are considerably higher than the expected MW for dodecamers, which could not be detected for any of the two constructs (S1 Table). Taking into account the likely association of small solution molecules, these species roughly matched to 21–24 or 28–30 combined monomers, respectively. Despite not always fitting to integer combination of hexamers, the fact that such species were only detected for the two CcmK2 constructs suggested the existence of potential CcmK2 pre-assembled intermediates in solution. Ion mobility spectrometry (IMS) experiments were therefore performed to evaluate whether the shape of these objects fitted better to sheet-like intermediates or to globular aggregates. Collision cross-sections (*Ω*) determined for selected K2^-^ and ^-^K2 species were slightly higher than values measured (and published) for globular proteins of similar MW (S4 Fig). Although this difference could be considered evidence for small layered assemblies, the absence of a continuous distribution of oligomers spreading up to higher m/z values, where Ω differences between sheet-like viral capsid proteins and globular structures are more notorious, limited the interpretation of CCS data.

### CcmK2 and CcmK4 assemble as flat sheets on mica

**Fig 2:**
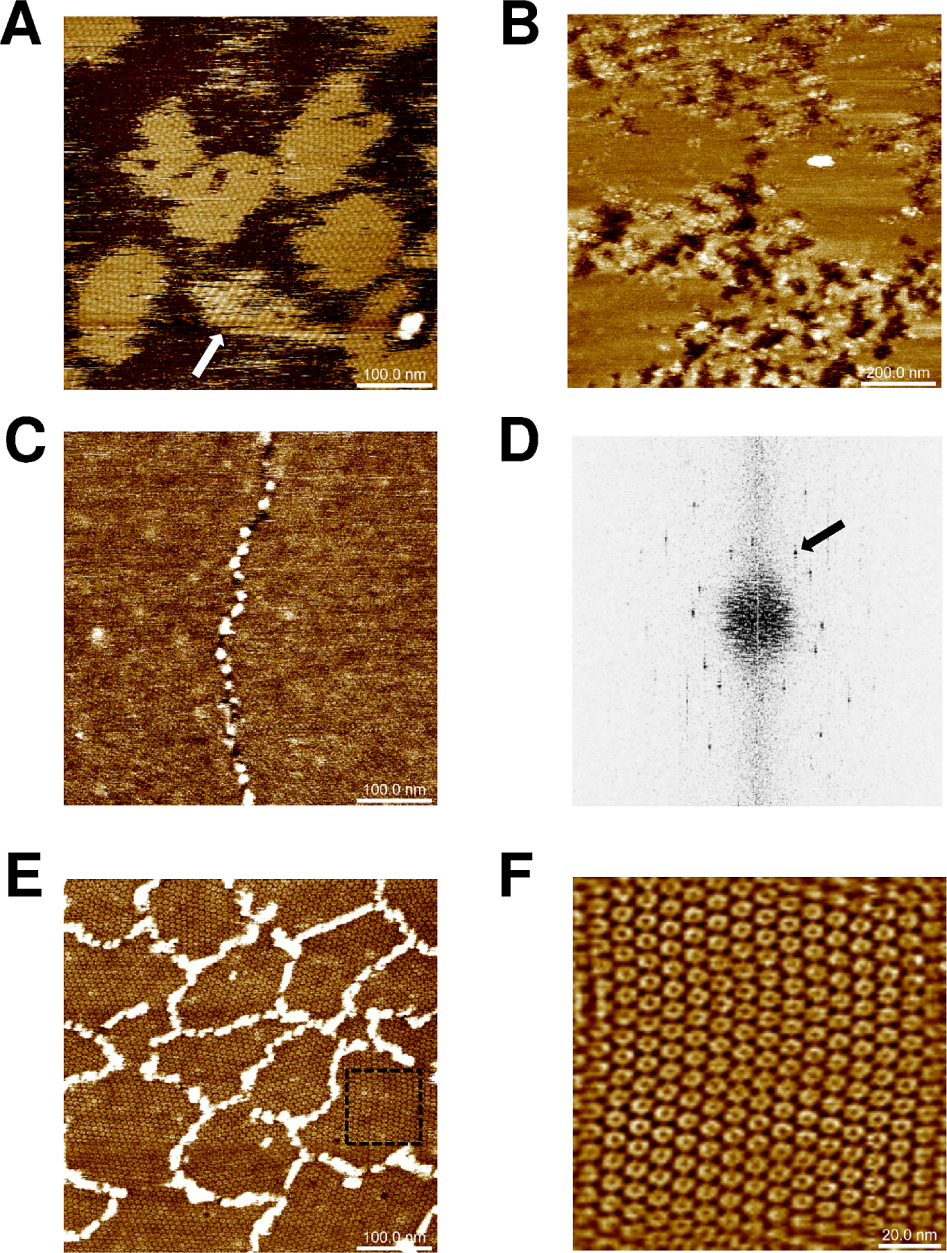
CcmK2 and CcmK4 assembling revealed by AFM. Images were recorded after absorption on mica of indicated amounts of proteins in phosphate buffers: 120 ng of K2^-TH^ at pH 6.0 (panel *A)*, 60 ng of ^HT-^K2 at pH 7.5 (*B*) and 120 ng of ^HT-^K4 at either pH 7.5 (*C*) or pH 5.5 (*E*). Stripped patches (arrow) were occasionally observed with K2^-TH^. Panel *D* results from applying FFT on image C. The black arrow indicates a periodicity spot at 6.9 nm. In *C* and *E*, ^HT-^K4 islands are surrounded by connected stretches of protein positioned at higher level. *F*, image obtained after inverse-FFT using periodicity spots generated from a cropped section of image from panel E (dashed square). Image sizes were five times the dimension of scale bars, 120x120 nm for panel F.

Assembly of ^HT-^K2, K2^-TH^ and ^HT-^K4, K4^-TH^ on mica supports was next investigated by atomic force microscopy (AFM). The influence of pH (from 5.0 to 8.0, 0.5-unit intervals), buffer composition (phosphate, MES or Tris) and protein concentration (20–120 μg/mL) was systematically evaluated. Extended islands of sheets were obtained when 0.4 μM concentrations of K2^-TH^ were deposited in phosphate (Fig 2A) or MES buffers at pH 6.0. Cross-sectional analysis proved the formation patches made of single protein layers (average depth of 4.1 ± 0.1 nm). Sheets were flat and seemed to derive from hexamers adopting a single orientation with regard to the mica plane. However, islands displaying striped motifs were also noticed in rare instances (arrow, Fig 2A), providing evidence for some degree of assembly plasticity for this isoform. Strips were 12–15 nm wide, with top plane lifted by about 1 nm above the lowest strip, which reached a similar height as other flat patches. Flat sheets were also imaged with ^HT-^K2. Assemblies persisted over a wider pH range, spreading from 5.5 to 7.0 (Fig 2B). The result was sharply influenced by protein concentration, small increases resulting in shift from no protein adsorption to fully covered mica. Difficulties to sense mica level impeded accurate estimations of layer thickness with ^HT-^K2 (4.8 ± 0.2 nm for data of Fig 2B). Intercalated patches reminiscent of assemblies bound to mica with inverse orientation were occasionally noticed, reaching 0.5 to 1 nm higher levels than the first level layer.

Similar experiments were carried out with ^HT-^K4 and K4^-TH^ proteins. Well resolved images recorded for ^HT-^K4 confirmed the formation of flat assemblies under a variety of conditions, with pH spreading from 5.5 to 7.5 (Fig 2C–2F). Likewise CcmK2, layer heights could not be accurately measured due to difficulties to prevent full covering of mica. An underestimated value of 3.1 ± 0.8 nm was calculated from an image recorded at pH 6.0. Fast Fourier transform (FFT) operations on images acquired at pH 7.5 revealed the occurrence of 6.9–7.1 nm periodicities (Fig 2D), matching values of 2D layer lattices from most of published 3D structures (approx. 70 Ǻ) [23, 35], but slightly longer than 6.7 nm values reported between hexamers interacting in a planar geometry within the HO BMC shell [26], or in crystals of tightly packed CcmK2 and CsoS1A proteins [32, 33]. Much like in EM studies ^13^, we could not detect patterns with strips of inversely oriented hexamers characterized in a CcmK4 crystal structure [35]. Less compact sheets formed at pH 5.5 (7.0 and 8.0 nm periodicities, depending on lattice direction), hexamers being held together seemingly through vertex-vertex contacts (Fig 2E and 2F). This organization contrasts with edge-to-edge interactions that must occur at pH 7.5, according to lattice periodicities similar to published crystal and EM data. Only at pH 5.5, central depressions at the center of hexamers were noticed, suggestive of assemblies deposited on mica with the opposite orientation. At all pHs, stretches of proteins were found decorating island edges, protruding in average 4 to 5 nm above the first layer level (Fig 2C and 2E).

Unfortunately, experimental conditions compatible with assembly of K4^-TH^ could not be found, something that we attributed to the proteolytic sensitivity of this construct that was confirmed in n-MS experiments (see S3B Fig).

### Honeycomb-like supra-hexameric organization of CcmK1 assemblies

**Fig 3:**
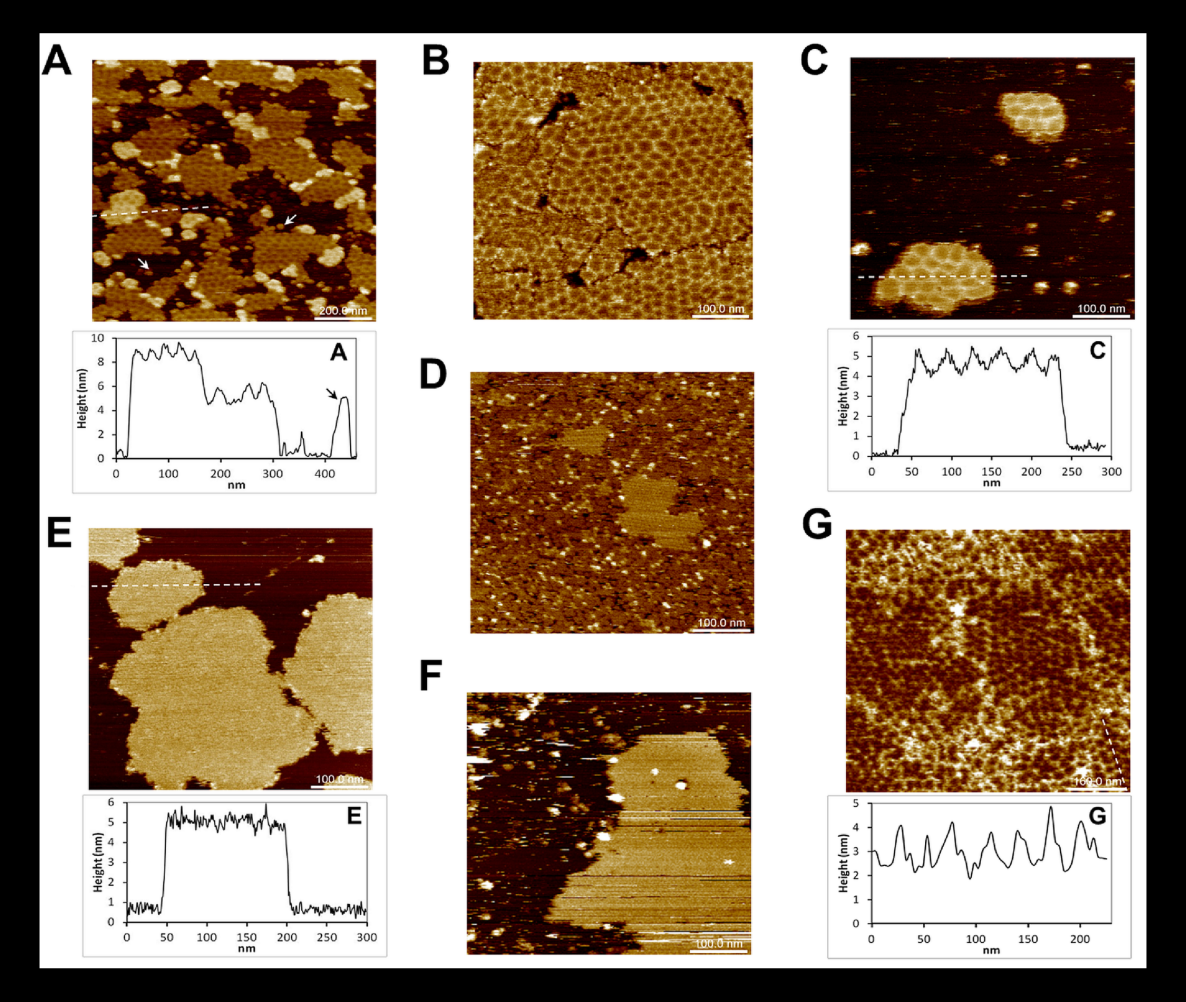
Supra-structural organization of CcmK1 from *Syn sp*. PCC6803. AFM images were recorded after absorption on mica of 100 ng of K1^-TH^ 6803 (*A*,*B*), 65 ng of K1^-H^ 6803 (C), 12 ng of ^H-^K2 7942 (*D*), 12 ng of K2^-H^ 7942 (*E*), 500 ng of untagged K1^-^ 6803 (*F*) or 50 ng of K1^-ncTK^ 6803 (*G*). Experiments were performed in either MES pH 6.5 (*D*), NaPi pH 6.5 (*B*, *C*, *E*, *F*, *G*) or TRIS pH 7.0 (*A*). Arrows in panel A indicate structures of size and thickness comparable to individual polygonal tiles. For panels A and F, solutions were supplemented with 0.5 mM guanosine-5′-tetraphosphate (GtetraP) and 5 mM MgCl_2_, respectively. Cross-sectional analysis graphs indicate material thicknesses traced along discontinuous white lines on images. Image sizes are 5 times those of indicated scale bars.

The propensity of K1^TH^ to self-organize on mica was next inspected. As for CcmK2 and CcmK4, experimental conditions were systematically screened. Sheets formed under varied conditions, yet the best resolved images were recorded in the pH 6.5–7.0 interval. Remarkably, K1^TH^ displayed a supra-hexameric level of organization resulting in honeycomb-like motifs (Fig 3A and 3B), contrasting with data presented for CcmK2 and CcmK4. Depending on the conditions, these motifs occurred within isolated islands (Fig 3A) or formed continuous assemblies almost fully covering the imaged surface (Fig 3B). These mosaics were detected not only growing directly on top of the mica support (Fig 3A) but also as stacked layers positioned above similarly honeycomb-structured material (Fig 3A) or even over a first layer of protein not displaying these peculiar motifs (S5E Fig). Heights of 4.8 ± 0.2 nm and 8.3 ± 0.3 nm with regard to mica were estimated for the first and second layers from Fig 3A, respectively. Values of *ca*. 7.2 nm were estimated from Fig 3B, despite difficulties to sense mica level at such high surface occupancy.

In spite of an evident heterogeneity of size and shape, average motifs might be described as polygons, approximately 25 to 35 nm large. A mean surface of 720 ± 90 nm^2^ was estimated from visually-selected representative cases. Since the largest face of a CcmK1 hexamer covers approximately 45 nm^2^ in the crystal structure[33], honeycomb tiles should contain in average at least 16 hexamers, depending on the curvature. Such structures were limited by edges that protruded above their central depression by roughly 1.2 to 1.5 nm. These values are well below CcmK hexamer thickness of about 3.5 nm. Thus, the contours cannot be explained as stacks of protein positioned on top of a flat plane of CcmK1. Heterogeneity was accompanied by variability of cross-section profiles (shown in Fig 3). It is thus not possible to conclude anything about the form of these patches, which might be either V- or round-shaped, or a mixture of the two. For some polygons, the edges progressively rose over the full path from the center (approx. 15 nm). In contrast, other motifs were basically flat and edges protruded sharply, over the size of a single hexamer (7 nm). These two situations would roughly correspond to 5°-12° bending angles distributed between a few hexamers within the same section of round-shaped patches, or 10°-24° at sharp edges of V-shaped patches. Noteworthy are spots of size and thickness comparable to individual polygonal tiles that were regularly detected under different set of conditions (e.g. arrows in Fig 3A), especially at low protein concentrations. However, the formation of honeycombed mosaics only occurred when negatively charged phosphate/phosphate esters or sulfonates were present, either as additives or buffer, during mica adsorption. Other additives assayed, including sulfate and bicarbonate salts or divalent cations, did not promote the formation of polygon-tiled assemblies (S5 Fig).

Overall, these data suggest that CcmK1 of *Syn6803* might play a role different from that ascribed to CcmK isoforms for CB assembly. Such behavior was not unveiled in 3D and EM structures, which pointed to similar (canonical) flat arrangements for the three isoforms, the single exception being the striped pattern of CcmK4 in 3D crystals[35]. We therefore questioned whether this apparent discrepancy for CcmK1 could derive from alterations caused by studied protein constructs or by different experimental constraints. These possibilities were tackled by studying K1^-H^, the construct used in previous structural investigations [23, 33]. Assemblies of purified K1^-H^ formed under similar experimental conditions than with K1^-TH^, and more importantly giving rise to the same supra-structural motifs (Fig 3C). Polygons were heterogeneous, yet of similar size than those produced by K1^-TH^ (average 700 ± 100 nm^2^ area). Surrounding edges protruded *ca*. 1 nm above the central depression. Assembled islands were positioned on average 4.3 ± 0.1 nm above mica, closer than values measured for K1^-TH^ islands (Fig 3A). The difference could arise from the presence of a 9 residue-longer linker preceding oligohistidine tags in K1^-TH^, as compared to K1^-H^ (S1 Fig), and argues in favor of an assembly model with tags mediating attachment to mica.

Two other CcmK1 variants were studied to shed light on the role of tags. First, untagged K1^-^ was prepared by TEV protease treatment of K1^-TH^. The resulting protein failed to assemble on mica, irrespective of protein amounts (60 to 500 ng), buffer composition (phosphate, MES, Tris) or pH (6.0 to 7.0, 0.5-unit intervals). Assembled islands could finally be produced at 8 times higher protein loads than with tagged proteins, and only in the presence of magnesium ions, conditions that resembled those applied in the AFM study of HO BMC-H [39]. Assembled islands of K1^-^ were flat, without apparent higher order motifs (Fig 3F). Heights above mica plane were estimated 3.7 ± 0.2 nm, similar to 3.5 nm values reported for untagged HO BMC-H [39], and in agreement with a closer proximity to the mica plane than with tagged K1^-TH^ or K1^-H^. The second variant differed from K1^-TH^ by the replacement of His_4_ by Lys_3_ tags, which in virtue of their positive charge should still ensure attachment to anionic mica. This protein K1^-ncTK^ was obtained by TEV treatment of a purified CcmK1-ncTEV-Lys_3_-TEV-His_4_ construct (K1^-ncTK-TH^), with ncT standing for “non-cleavable TEV peptide” (S1 Fig). Assembly of K1^-ncTK^ on mica occurred under similar conditions than with K1^-TH^, and most importantly giving rise to comparable polygonal mosaics (Fig 3G). Differing from K1^-TH^ and K1^-H^, however, arrangements reaching two slightly different heights were noticed. Polygone edges also protruded to higher levels (up to 2 nm) above their center than K1^-TH^ or K1^-H^ (cross-section profile of Fig 3G).

AFM data for K1^-TH^, K1^-H^ or K1^-ncTK^ were not well enough resolved to define unambiguously the contour of assembly units. Efforts to enhance image definition by combining series of successive scans covering the same region failed. Comparison of five aligned images taken at 15 min intervals evidenced continuous size and shape alterations of polygonal patches, indicative of dynamic fluctuations of the assemblies (see below), or alternatively of perturbations induced by the AFM tip. With the same intention, we attempted the imaging of 2D sheets after cross-linking reactions with glutaraldehyde. Unfortunately, the low protein coverage detected on mica indicated disruption of the assemblies by the dialdehyde.

### Assembly dynamics

The dynamics of K1^-TH^ assembly was next investigated by high speed (HS-)AFM. This approach was applied recently to characterize how flat 2D sheets form from individual HO BMC-H that are mostly recruited to the edges of assembled islands [39]. Two experiments were mounted, addressing either the characterization of assembly formation or the dynamics of pre-formed suprahexagonal motifs. To monitor the assembly process from its beginning, the protein was injected once the AFM probe already engaged in contact with the mica surface. Frames were recorded every 4 sec for 1 hour period. In that manner, the formation of the polygonal tapestry made of K1^-TH^ could be monitored (S1 Movie and S6 Fig). Despite the fact that image quality was limited, recorded data demonstrated that the assembly formation on mica is a dynamic process. Continuous events of attachment and detachment of individual proteins occurred during the first minutes (S6 Fig, frames 1 to 4). This was followed by the appearance of small seeds (frames 5 to 8), some anchored to the surface, and the later emergence of patches reminiscent of individual curved polygonal structures (frames 9 to 12). Later stages revealed a tendency for the curved domains to serve as template and boost the emergence of new neighbor curved structures, the ensemble finally coalescing into honeycomb mosaics like those presented in Fig 3A–3C (frames 13 to 16). Data indicated tendency for the HS-AFM data also demonstrated that polygonal motifs are continuously remodeled both in size and shape.

For the second experiment, a polygonal tapestry of K1^-TH^ was allowed to form prior to scanning the surface at 10 sec intervals for 20 minutes. Comparison of aligned frames demonstrated that most active regions for sheet remodeling are assembly edges, as proven by higher standard deviations measured at such emplacements when comparing the series of aligned images (S2 Movie and S7 Fig). Structural fluctuations were also noticed at edges of honeycomb polygonal motifs, although to a lesser extent than was observed for the island edges. Overall, these data demonstrated the suitability and weakly invasive character of AFM approaches. Visualization of attachment and detachment events of oligohistidine tagged CcmK1 on mica supports gave additional credibility to the significance of the structural motifs characterized herein.

### Honeycomb supra-hexameric organization of CcmK1 disrupted by single point mutations

Residues K25, R28, D49 and R80 are estimated by theoretical algorithms to be major contributors to inter-hexamer interactions. Their role in promoting high-order assembly was therefore investigated. Residues were mutated either individually or in combinations of two (with the exception of the K25A/R28A, which was not attempted). Unfortunately, although well expressed, none of the proteins incorporating the K25A mutation could be recovered in IMAC-purified fractions (arrows, S8 Fig), contrasting with a 10-fold increased solubility over WT protein, reported for the corresponding amino acid exchange variant K26A of a PduA [43]. Since substitutions of charged residues were expected to shift the isoelectric point of hexamers and thereby could influence attachment to mica, AFM studies were carried out with each variant at pH values ranging from 5.5–7.0 (0.5-unit intervals). The supra-structural assemblies characterized for K1^-TH^ (and K1^-H^) were disrupted in R28A or R80A single mutants and in the R28A/R80A double mutant (S9 Fig), which remarkably continued to assemble. The three mutants gave rise to islands of flat sheets, resembling those observed with CcmK2 and CcmK4. In contrast, the D49A mutant displayed a trend to produce higher-order assemblies reminiscent of honeycomb polygonal motifs described above for WT protein, yet with much more irregular sizes and shapes (S9B Fig). Finally, the double mutant D49A/R80A did not result in any identifiable organized pattern on mica (S9E Fig).

### Residues on the second α helix and at the C-ter extension contribute to CcmK1 curvature

Only seven residues and the presence of a short C-terminal extension distinguish CcmK1 from CcmK2 of *Syn6803* (see S10A Fig). Comparison of the two protein structures indicates that, apart from the flexible C-terminal extension, the most diverging region localizes at the C-terminus of the second α-helix where three consecutive diverging residues accumulate (S10B Fig). The calculated root mean square deviation (RMSD) of coordinates for the 24 backbone atom (ba) of residues 61 to 66 is 1.55 Å. For comparison, RMSD of 0.51 Å were measured for the remaining 344 ba in between residues 3 and 94 of the two proteins. Similar differences are found when comparing the structure of CcmK1 *Syn6803* and those of CcmK2 from *Synechococcus elongatus PCC 7942* (hereafter *Syn7942*) (1.45 vs. 0.33 Å) or CcmK4 *Syn6803* (1.52 vs. 0.69 Å).

The possible implication of this α2 short helical segment and of the C-terminal extension in the formation of assemblies displaying supra-hexameric motifs was first examined in AFM experiments with CcmK2 *Syn7942*. N-ter His4-tagged (^H-^K2 *Syn7942*) and C-ter His6-tagged (K2^-H^ *Syn7942*) versions were purified and their organization on mica inspected. Both proteins showed a clear propensity to form flat 2D sheets (Fig 3D and 3E), in agreement with sequence and structural similarity to CcmK2 *Syn6803*. Assembled patches of ^H-^K2 *Syn7942* displayed two orientations with regard to mica surface, whereas a single orientation was observed for K2^-H^ *Syn7942*. Average layer thickness over mica measured for the latter was 4.2 ± 0.2 nm, and approximately 2.4 and 3.3 nm for the two ^H-^K2 orientations, although these values were undoubtedly underestimated bearing in mind the difficulties to sense mica. Supra-hexameric organizations were not detected in any of the conditions screened, which covered variable protein concentrations, buffers, and pHs ranging from 6.0 to 8.0.

**Fig 4:**
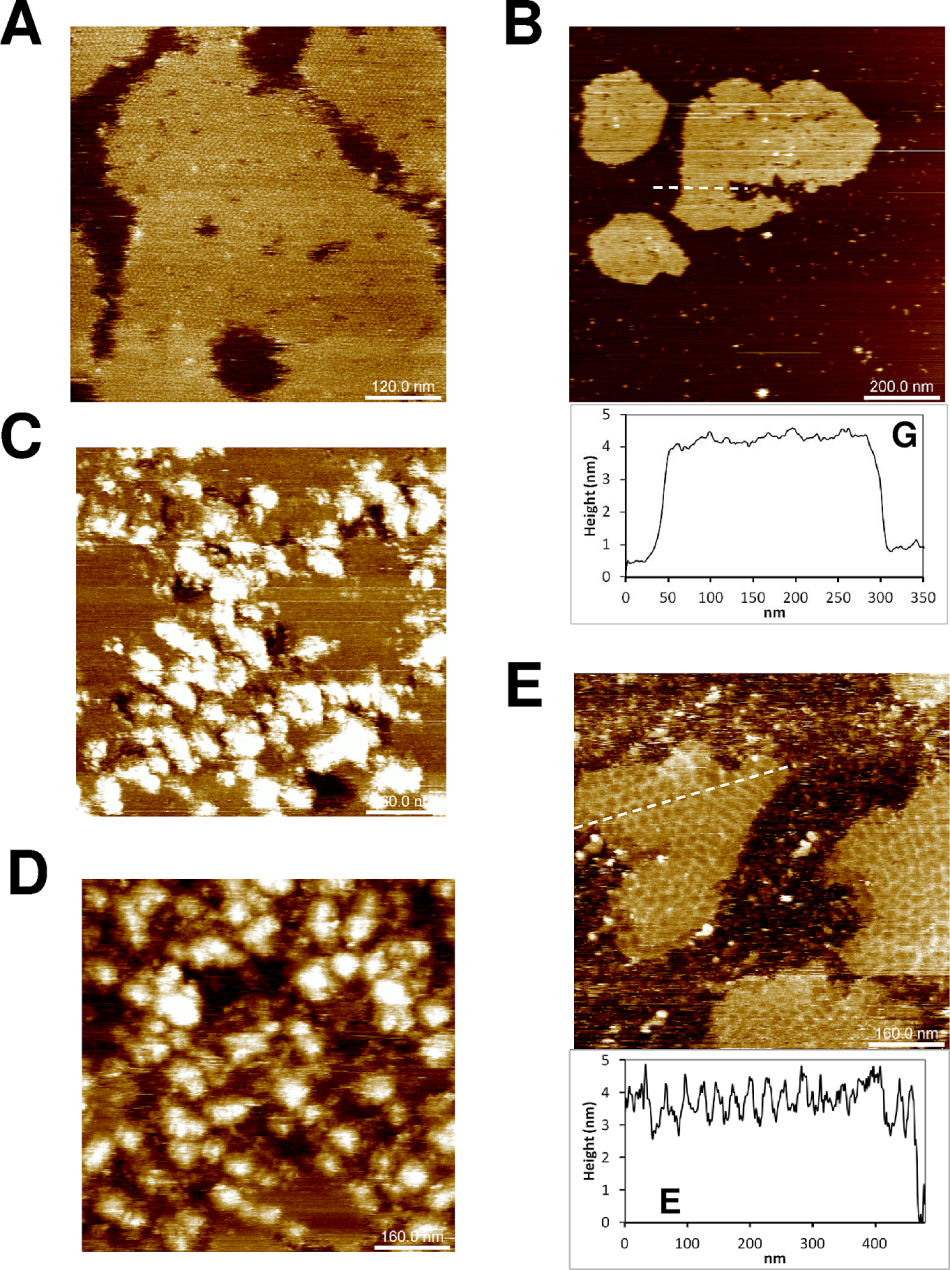
Residues of the second a helix and the C-ter extension of CcmK1 from *Syn sp*. PCC6803 are required for formation of polygonal mosaics. AFM images were recorded after absorption on mica of 100 ng of A^63^AN K1^-TH^ 6803 (*A*), 100 ng of D9 K1^-TH^ 6803 (*B*) and 45 ng of either N^63^IR K2^-TH^ 6803 (*C*), or 9Cter K2^-TH^ 6803 (*D*), or N^63^IR/9Cter-K2^-TH^ 6803 (*E*). Experiments were performed in either MES pH 6.5 (*B*, *D*, *C*) or NaPi pH 6.5 (*A*, *E*). For panel E, the solution was supplemented with 0.5 mM GtetraPi. Cross-sectional analysis graphs indicate material thicknesses traced along discontinuous white lines on images. Image sizes are 5 times those of indicated scale bars.

To further uncover the participation of the diverging α2 helix stretch and/or of the last C-ter residues in generating CcmK1 *Syn6803* honeycomb mosaics, two new K1^-TH^ constructs were prepared: i) AAN K1^-TH^: the triple mutant with N^63^I^64^R^65^ replaced by the corresponding AAN residues of CcmK2 *Syn6803*; ii) Δ9 K1^-TH^: protein with the last 9-residues deleted (blue bar, S10A Fig). The two proteins were purified and studied on mica. Protein AAN K1^-TH^ resulted in flat sheets placed at 3.9 ± 0.3 nm above the mica plane (Fig 4A), when assembly was allowed to occur in the presence of phosphate or phosphoesters. Something similar was noticed with Δ9 K1^-TH^, the protein tiling at about 3.4 ± 0.1 nm heights in the presence of MES or NaPi buffers (Fig 4B).

Three CcmK2 *Syn6803* variants were engineered, purified and assayed with the intention to further confirm the implication of the α2 helix stretch and the C-terminal extension to the peculiar assembly behavior of CcmK1: i) NIR K2^-TH^: the triple mutant with A^63^A^64^N^65^ replaced by the corresponding NIR residues of CcmK1 *Syn6803*; ii) 9Cter K2^-TH^: CcmK2 protein incorporating the last 9-residues of CcmK1; iii) NIR/9Cter K2^-TH^, which combined the two modifications of CcmK2. The first two proteins gave rise to flat assembled patches (Fig 4C and 4D), often contoured by aggregated material. In agreement with the observations described above, supra-hexameric polygonal motifs only formed when the two elements were integrated in CcmK2 (Fig 4E). Overall, these data convincingly evidenced the combined participation of the two structural elements to the formation of honeycomb mosaics observed with wild-type CcmK1.

### *In silico* simulations of CcmK assembly

**Fig 5:**
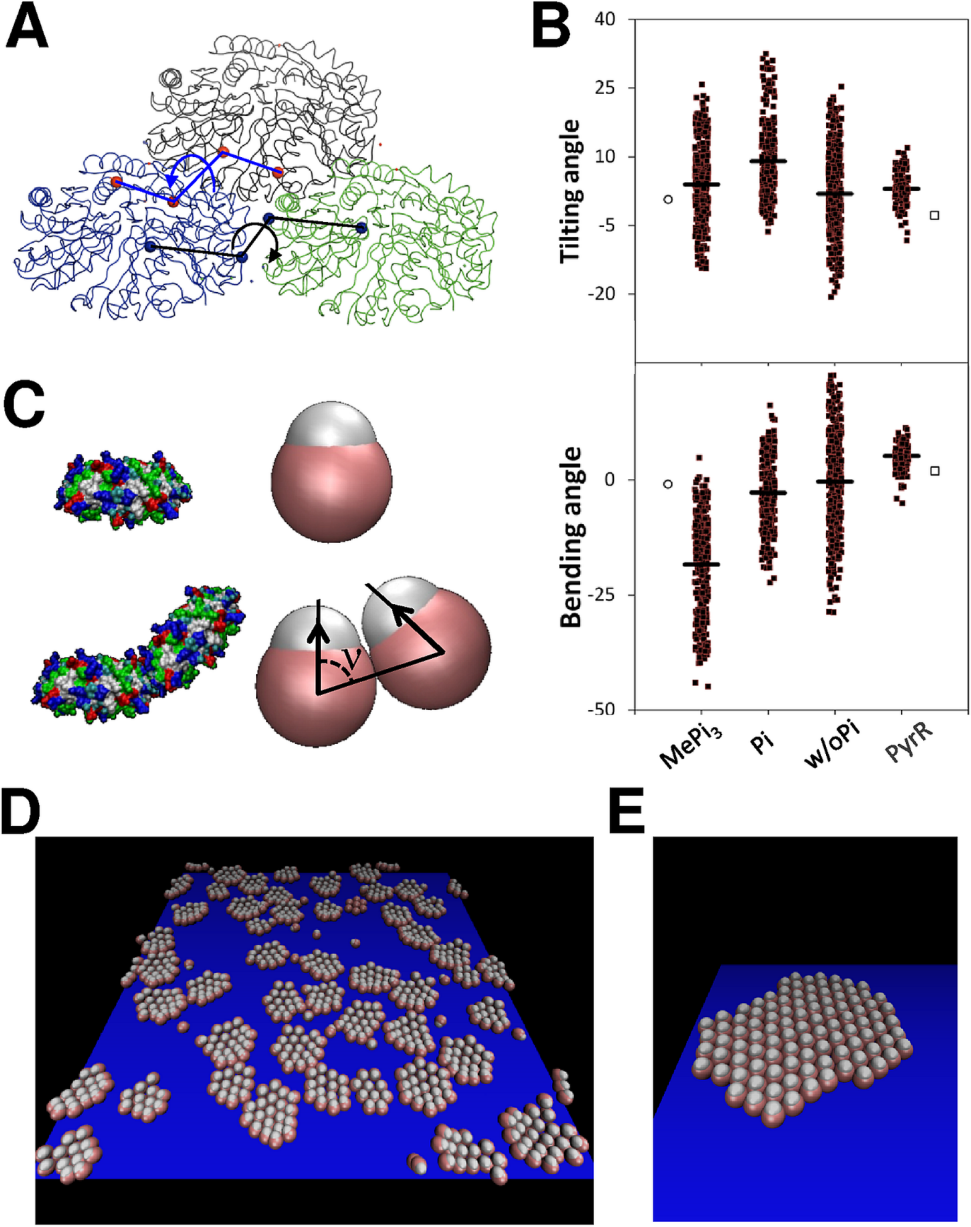
Theoretical simulations of CcmK assembly. *A-B*, All-atom molecular dynamics of an ensemble of three CcmK1 *Syn6803* hexamers originally positioned as in the published crystal structure (PDB code 3BN4). *A*, Ribbon representation of the simulated system (excluding explicit water and salt molecules). The position of atoms selected for measurement of tilting and bending angles are indicated with red and blue spheres, respectively (only shown for a single couple of hexamers for clarity). Tilting is defined by main-chain alpha carbon (Cα) atoms from residues 23 and 17 from interfacial monomers in each hexamer (illustrated by blue arrow). Bending angle values were based on the positions of Cα atoms from residues 26 and 37 from interfacial monomers (black arrow). *B*, Plot of tilting and bending angles during all-atom MD simulations (30 ns), measured at 0.25 ns intervals (black squares). Three different starting models were investigated, depending on the absence (w/oPi) or presence of Pi or MePi_3_ ligands replacing crystallographic sulfates at the interface between hexamers. Shown are deviations from approximate 180° measured for the original crystal structure (empty circles). Thick traces represent mean values over the MD run. Similar data measured during a similar 30 ns simulation with a PyrR dimer (PDB code 1A4X) are presented for comparison. Here, tilting is defined by Ca atoms from residues 100, 104 of chain A, and 105, 131 of chain B, whereas bending angle is read from Cα atoms from residues 104, 143 of chain A and 143, 103 of chain B. Angles measured from the starting crystal structure are presented as empty squares. *C*, Lateral views of CcmK1 structure (*left*), and its representation in the coarse-grained model (*middle*). The white sphere indicates hexamer orientation. Two hexamers assembled with a nonzero curvature are illustrated below, indicating the preferred angle ν of interaction (bottom). *D*, snapshot of a coarse-grained simulation run with parameters ε_0_ = 8.3 *k*_*B*_*T*, *ν* = 1.4 rad, *ε*_ads_ = 2.5 *k*_*B*_*T*, *α* = 0.1 and hexamer concentration 0.0125 σ^*-3*^. The solid support plane appears blue and only hexamers near the surface are plotted. The simulations show the presence of stable patches of 15–20 hexagons with raised borders that resemble structures formed during the assembly of Ccmk1 with phosphate/sulfonate anions. *E*, snapshot of a coarse-grained simulation run with parameters ε_0_ = 6.7 *k*_*B*_*T*, *ν* = 1.45 rad, *ε*_ads_ = 10 *k*_*B*_*T*, *α* = 0.2 and hexamer concentration 0.0125 σ^*-3*^, showing the formation of a canonically flat assembly, that occurs even with non-flat preferred curvatures at weak bending stiffness and high adsorption strengths.

CcmK1 assembly behavior was investigated by atomistic molecular dynamics (MD). Simulations were launched on ensembles of three interacting hexamers, initially positioned as observed in the 3D crystal structure (PDB code 3BN4). The original structure included sulfate molecules modeled at the positively charged site found at the twofold axis of symmetry, where two hexamers meet [33]. Taking into consideration the requirement for phosphate/sulfonate molecules to attain curved assemblies in our experiments, the importance of this site was inspected as well. Thus, interfacial sulfates were either removed (w/o Pi), or replaced by inorganic phosphate (Pi) or methyl-triphosphate molecules (MePi_3_) for MD simulations. These simulations clearly evidenced weak or no preference for flat structures under any condition. Thus, interhexamer tilting and bending angles measured for intermediate structures throughout MD trajectories deviated rapidly from values measured for the 3D crystal structure (Fig 5A and 5B, S3–S5 Movies). Most notable distortions were noticed for the interhexamer bending angle with CcmK1:MePi_3_, values shifting on average by 17° with regard to the crystal structure (Fig 5B, S3 and S4 Movies). Moreover, a clear tendency of hexamers to curve towards the same side was revealed with this ligand (convex face towards the interior of the curved assembly), in contrast with data from simulations with Pi or w/o Pi assemblies (S5 Movie), which indicated bending in both directions and average values close to the starting planar situation. A closer inspection of engaged interactions suggested that ionic contacts between R66 side-chain and phosphates of MePi_3_ could be a determinant in holding bent structures (S4 Movie). Interhexamer tilting was also manifested, yet average values remained close to starting values, the value shifting by about 8° in simulations with sandwiched Pi molecules. Concerning interhexamer distances, average value decreased with MePi_3_ by about 0.4 Ǻ with regard to the 3D structure, whereas the value augmented slightly by 1.1 and 1.6 Ǻ for Pi and w/o Pi structures, respectively. Contrasting with CcmK1 simulations, higher conformational rigidity was revealed by similar MD simulations on a dimer of a *B*. *subtilis* operon repressor of pyrimidine biosynthesis (PyrR) [44] (Fig 4B, S6 Movie).

The formation of larger assembled patches was next simulated using a minimal coarse-grained model. This is a well-established tool for investigating complex assembly processes [31]. Although structure-function relationships remain inaccessible, these models permit to parse biophysical constraints that might influence the final fate of such complex processes. As remarkable demonstration, Perlmutter *et al*. recently defined energetic regimes and conditions that lead to empty or cargo-filled BMC [31]. In our model, hexamers were represented as nearly spherical beads interacting with a simple potential that included: a short-range repulsion term that prevents hexamer collapse; an attraction term driving the assembly; bending and torsion terms accounting for the preferred angle of interaction between hexamers and the energetic cost of bending and twisting them from the optimal angle (more details are provided as M&M and ref [45]). Based on this model, Brownian dynamics simulations successfully reproduced the formation of assembled patches of CcmK1. Depending on selected relationships of parameter values, either flat patches or curved assemblies formed (Fig 5C and 5D and S7 Movie). Curved patches reminiscent of those found in honeycomb-like structures, composed by 15–20 assembly units, formed when the preferred interhexamer interaction angle was not flat. Despite the fact that assembly could occur in solution or from surface-attached hexamers, the survival of curved patches on the flat support required the imposition of weak adhesion forces, supporting our experimental working choice of mediating contacts with mica through oligohistidine tags.

## Discussion

Genomic data mining proved the presence of variable numbers of BMC-H, BMC-T and BMC-P paralogs, with a minimum of four components in species carrying less complex micro-compartments [1]. Among them, BMC-H proteins are assumed to be the main constitutive bricks of flat icosahedral CB facets. Basically, the consensual assumption of flat BMC-H tiling in facets was based on abundant crystal structures that confirmed the propensity of hexamers to assemble side-by-side into tightly packed 2D sheets, an observation further reinforced by EM data [23, 46]. CcmK proteins were therefore ranged among components of shell triangular facets. Conversely, AFM data presented in this work indicate that CcmK1 could play an alternative, or most likely an additional structural role. Instead of forming canonical flat sheets, *Syn6803* CcmK1 assembled into a polygonal tapestry made by patches of *ca* 30 nm diameter with borders detached from the surface and elevated by about 1–1.5 nm with respect to their center. Such patches formed directly on mica (Fig 3A), but also above structured (Fig 3A and 3B) and apparently unstructured protein layers (S5E Fig), pointing to a manifestation of an intrinsic property of this isoform. Isolated assembled islands composed of such polygonal mosaics were also characterized, ruling out the possibility of distortions arising from protein crowding effects on the support. Moreover, the dynamics of formation of such curved patches could be successfully monitored by HS-AFM. Albeit unexpected, a few reported antecedents exist for non-flat and rounded BMC-H assemblies: most importantly, planar and 30°-bent hexamer-hexamer geometries were found to coexist within the 3D structure of *HO* BMC shells [26]; tubular structures of PduA forming *in vivo* and characterized by TEM were interpreted as piled curved assemblies made of 30°-tilted hexamers [34]; rolled-up sheets of HO BMC-H were also imaged directly from cells by EM [39], contrasting with canonical 2D arrays characterized for the purified protein [46]. We notice, however, that the orientation of curved assemblies characterized here, with convex side facing the interior of curved patches (see below), appears to be incompatible with the expected cargo-shell scaffolding mode, if interactions between CcmK1 C-terminal helix and the CcmN targeting peptide occurred following the pattern reported for Pdu BMCs [47]. Conversely, our data is in agreement with the inversed orientation of BMC-H components observed in *HO* BMC shells [26], suggesting that shell motifs recognized by targeting peptides might differ between BMC types.

Selecting to study tagged constructs was decisive in revealing CcmK1’s behavior, probably for the characterization of stripped assemblies with CcmK2 too. Oligohistidine N- or C-ter tags, both located on the concave face, facilitated adsorption on mica, as evidenced by the difficulties to image the untagged protein K1^-^, by the usual observation of homogenously-oriented hexamers and the fact that protein deposition augmented considerably at pH < 7.0, which is close to *pK*a of histidine side-chain, also by the increase in heights of assemblies with regard to mica in the order K1^-^ > K1^-H^ > K1^-TH^. In mediating interactions with mica, oligohistidine tags lifted the assembly plane above the support, something expected to increase conformational freedom and to relax constraints induced by direct interactions with the support. Alternatively, oligohistidine tags might be argued to modify CcmK1 behavior, especially considering that flat assemblies were visualized for the untagged protein. An argument against this possibility is that canonical flat sheets were produced with many other K1^-TH^ variants/mutants, also with K2^-TH^, all of them sharing the same C-ter sequence stretch. Moreover, curved polygonal mosaics also formed when oligohistidines were replaced by a Lys_3_ polycationic tag. Indeed, the dissimilar behavior uncovered for an identical CcmK1 construct (K1^-H^) by AFM and EM can only be justified as being caused by different experimental approaches^19^. Coincidently, Dryden *et al*. noticed differences between CcmK1 with regard to CcmK2 or CcmK4 isoforms in those EM studies. The former was more readily characterized as single monolayers, whereas CcmK2 and CcmK4 tended to form superimposed 2D crystals.

Spots resembling individual assembled patches were observed, with low resolution, for CcmK1 *Syn6803* under varied conditions throughout our study. However, holding the patches together into larger assemblies (Fig 3A–3C) was dependent on the presence of phosphates or sulfonates. Interestingly, electron densities compatible with such anions were revealed in several crystal structures of BMC-H, at the twofold axis of symmetry where hexamers meet to form layers [e.g. CcmK1 (3BN4, 3DN9, 4LIW), CcmK2 (3CIM), CsoS1A (2G13), or EutM (4AXJ)] [32, 33, 48, 49]. Such potential anion-binding sites are surrounded by cationic residues (i.e. K25, R80, R66, H82, following *Syn6803* CcmK1 numbering) that together with residues from the neighboring symmetric hexamer build funnels with strong electrostatic positive potentials. Resulting interstices might serve as conduits for anionic metabolites through BMC shells, as suggested before [35]. Alternatively, such sites could constitute nodes for structural assembly/disassembly. Thus, assembly bending (and stiffness) would shift in response to ligand binding, as suggested by our all-atom molecular dynamics data, the process becoming sensitive to the overall cellular metabolic state (e.g. AMP/ATP ratio or polyphosphate stock). Intriguingly in this sense, CBs were found to lie close to polyphosphate (polyPi) bodies in EM studies [6]. In other study, significant increases of transcription of CB genes were measured when polyPi formation was impaired in cyanobacteria [50]. These last authors interpreted their observations as indicative of inefficient assembly of CBs when polyPi content is reduced.

Mutational strategies were applied to investigate the structural causes behind CcmK1 *Syn6803* behavior. Sequence and structural comparisons to most homologous isoforms, CcmK2 from *Syn6803* and *Syn7942*, permitted to localize two regions concentrating major differences: the stretch comprising residues 63–65 of the α2 helix and a C-ter extension present in *Syn6803* CcmK1. To our surprise, AFM data proved that both elements were required to reproduce the non-canonical CcmK1 assembly behavior. This was first demonstrated by the occurrence of flat assemblies with both AAN K1^-TH^ and ΔCter K1^-TH^ mutants, and further confirmed by the observation of curved assemblies when the two modifications were engineered together in CcmK2 (i.e. NIR/9Cter K2^-TH^ variant).

Structural analysis indicated that the outwards displacement of the CcmK1 α2 helix could be caused by the presence of a polar Asn63 followed by the bulky Ile64. The first residue is replaced by Ala in CcmK2 *Syn6803* and CcmK4 *Syn6803*, whereas in CcmK2 *Syn7942* an Ala replaces Ile64. The consequence is an altered disposition of side-chains of residues 65 and 66 (numbered 67/68 in CcmK4) (S10B Fig). Most notably, the guanidinium of R66 of CcmK1 *Syn6803* revolved towards the potential phosphate-binding site, something that might reinforce interactions with ligands, e.g. with (poly)phosphates. This possibility is supported by the molecular dynamics data (Fig 4). Interestingly, this displacement of the α2 helix C-terminus is shared by all three *Syn6803* CcmK1 structures (PDB codes 3BN4, 3DN9 and LIW), also by *T*. *elongatus* CcmK1 (3SSS). In the latter, a NV stretch replaced NI 63–64 residues of Syn6803 CcmK1. In contrast, the α2 helix movement is absent from all other CcmK2 and CcmK4 structures (2A1B, 3CIM, 3DNC, 4OX7, 2A10 and 2A18), which carry AA, SA or AI residues at corresponding positions 63–64 of CcmK1. The region is even further displaced in CsoS1A and CsoS1C structures, which both present CA residues at the emplacement of NI of Syn6803 CcmK1. Structural effects caused by the bound sulfate are unlikely, since the same ligand at the same site in CcmK2 *Syn6803* (3CIM) was not accompanied by the α2 helical displacement. Sequence comparisons prove that NI, NV or comparable SV and SI stretches are present in at least one BMC-H isoform in 42 out of 46 compared β-cyanobacteria species (S11 Fig). The ORF of the corresponding isoform was invariantly located at the main carboxysome loci [1]. Among exceptions, the succession of polar/hydrophobic bulky residues at positions 63/64 was missing in all three CcmK from *Syn*. *elongatus PCC7942*, something that casts doubts on the generality of this structural feature.

The implication of the C-ter extension of *Syn6803* CcmK1 in the formation of polygonal mosaics is intriguing. This motif is supposed to be flexible (i.e. undefined in crystal structures). In *Syn6803* CcmK1, this portion includes two consecutive arginines and is preceded by a glutamic-rich α helix, an organization that is reproduced in CcmK4 but not in CcmK2. Extensions with two or three arginines exist at the C-terminus of at least one BMC-H isoform in all compared β-cyanobacteria (S12 Fig), and indeed often accompany NI (or comparable) residues at positions 63–64. However, similar extensions are absent from BMC-H of α-CB of cyanobacteria or chemoautotrophes. The C-ter extension of *Syn6803* CcmK1 is preceded by an α helix that adopted two major dispositions in crystal structures: oriented towards the hexamer center [e.g. in CcmK1 (3SSS) and CcmK2 (2A1B, 3SSQ)] or towards its edge [in CcmK2 (4OX7), in CcmK4 (2A10, 2A18)]. Kerfeld *et al*. hypothesized on the different orientations found in CcmK2 and CcmK4 crystal structures as a possible explanation to the striped-2D-arrangements displayed by the latter [35]. The helix was also implicated in contacts between hexamers from piled 2D layers in CcmK2 crystals, probably also in CcmK1 [33, 41]. On the basis of small buried areas and variability of precise interactions, these contacts were presented as non-relevant artifacts. However, comparison of gel filtration and electrophoresis data for truncated constructs and WT proteins proved the propensity of C-terminal tails to engage contacts [33, 41]. Gel filtration data presented here, and most notably the detection of potential pre-assembled intermediates by n-MS might support this view for CcmK2. Interestingly, a double-layered sheet (and not dodecamers) was proposed as best model to fit FRET data collected with *T*. *elongatus* CcmK2[41]. No evidence was obtained for similar interactions occurring in solution with CcmK1 or CcmK4. We propose that this isoform-dependent behavior might arise from interferences of CcmK1 and CcmK4 Arg-rich C-ter extensions, which would fold onto the preceding α helix from the same monomer or from neighbors in the hexamer, thus hampering contacts with other hexamers in solution. Alternatively, constraints imposed in packed assemblies would favor inter-hexamer interactions between inversely-oriented C-terminal segments. AFM data presented for CcmK4 would support this hypothesis. Considering that the only flexible elements in CcmK4 structures are the first three N-ter residues and last 99 to 111 residues, the edge-to-edge bridges that apparently connect CcmK4 hexamers in Fig 2E and 2F should correspond to paired anti-parallel C-terminal segments acting as a flexible ‘molecular velcro’. Similar arrangements of ionic residues in flexible C-terminal extensions were also noticed in structures of CcmL orthologs, and were indeed proposed to be sites for holding contacts with shell neighbors [51]. In this study, FRET experiments suggested that CcmL and (fluorescently-labeled) CcmK2 from *T*. *elongatus* BP-1 did not interact in a one-to-one basis in solution. FRET signal was only detected at > 100 μM concentrations of CcmK2, largely exceeding those of CcmL. This was interpreted as support for a scenario with CcmL interacting with CcmK2 at rare defect points in a growing CcmK2 proto-shell, in agreement with the possibility that Velcro contacts could only be triggered after first preassembly steps. In this way, interactions between the C-terminal tails of CcmK proteins would reinforce CB shell contacts, much in the same way as flexible termini of certain viral capsid proteins participate as switches for distinct types of interactions in mature viral capsid [52–54]. Native-MS and AFM experimentation on C-ter truncated/mutated versions, also in the presence of short mono and bidentate competing peptides, should allow validating or ruling out this assembling scenario.

Coarse-grained dynamic simulations successfully reproduced the formation of curved CcmK patches *in silico*. Worth-mentioning, inter-hexamer interaction strengths applied in our theoretical model were comparable to regimes required to reproduce *in silico* the assembly of empty shells [31]. The occurrence of patches of finite size pointed to a geometrically-frustrated mechanism of assembly[55]. In this scenario, the assembly of a curved layer, favored by the non-flat preferred angle of interaction between hexamers, accumulates elastic stress that eventually impedes further growth. Curved assemblies appeared both in solution and on surfaces, provided that the preferred angle of interaction was not flat, and that moderately high bending stiffness and weak adsorption strengths were applied. The first choice might explain the apparent discrepancy between our results and the attainment of flat assemblies in coarse-grained simulations with *Syn6803* CcmK2, since hexamers were forced to reside on a plane for those simulations[30]. The characterization of flat assemblies with untagged K1^-^ could also support the conclusions of our simulations. Thus, in the absence of the poly-cationic anchoring tags, hexamer cores likely settled on mica, in agreement with the smaller heights over mica measured for K1^-^ assemblies, as compared to K1^-TH^ or K1^-H^. Presumably, such direct and more regular contacts would constrain resulting assemblies to remain flat.

The existence of spontaneous assembly curvature was supported by atomistic MD trajectories that indicated no preference for planar interactions, a likely consequence of small contact surfaces covered between wedged-shaped hexamers. For comparison, significantly less conformational flexibility was revealed in MD runs launched on a real protein dimer, the *B*. *subtilis* pyrimidine-biosynthetic operon repressor PyrR [44]. BMC-H interhexamer contacts are classified as biologically irrelevant by specialized algorithms such as DiMoVo, which was conceived to discern between specific and unspecific protein-protein interfaces revealed in crystal structures [56]. Indeed, surfaces buried between hexamers in 3D structures of BMC-H/T fall in the 400 to 1200 Ǻ^2^ range (per edge)[36, 37, 57, 58], well below 1700 Å^2^ threshold values for dimeric biological complexes [29]. In crystal structures of BMC-H proteins, 2D layers are basically held together through few ionic and hydrogen-bond interactions. Mutation of such residues often led to hexamers that continue to tile together. This is the case for R28A, R80A and R28A/R80A CcmK1 mutants presented here (S9 Fig), also for similar mutants studied before[34, 39], although in other instances mutation was found to impact critically the assembly [43]. Possibly in line with this argumentation, Kerfeld and colleges could reconstitute *in vivo* chimera shells with an α-carboxysomal component (CsoS1A) integrated within a β-CB shell [36]. Cooperative interactions with weak interhexamer specificity would explain the permissive assembly behavior noticed for same or homologous BMC-H in 3D and 2D structures, as presented in the Introduction [24, 32, 33]^,^[34–36]. Structural plasticity is confirmed by the description here of CcmK1 curved patches (contrasting with reported canonical structures), of flat and striped assemblies with CcmK2, of flat 2D sheets with CcmK4 that differ from striped arrangements described from 3D crystal structures [35], and most importantly, by the characterization of flat and bent interhexamer dispositions in the structure of HO-BMC shells [26]. The unique mechanical softness and flexibility of β-carboxysomes, revealed by AFM-based nanoindentation, could also in part originate from such plasticity [27].

Round- (or V-shaped) and striped CcmK motifs characterized here or before strongly suggest that, apart from building flat facets, BMC-H proteins may serve additional structural roles. The peculiar behavior of CcmK1 might be important during CB biogenesis, by for instance permitting to adjust the assembly process to the extent and regularity of adhesion contacts to its support. Round patches might grow in early stages above partially-disordered procarboxysome seeds [16], or wrap around biogenesis intermediates giving rise to fragmented substructures like those observed before [6, 27]. Interestingly, Perlmutter *et al*. found that the formation of closed BMC was more robustly reproduced in coarse-grained simulations when seeds were allowed to be partially fluid (i.e. non crystalline) [31]. An evolution towards planar facets would then accompany the establishment of more regular contacts with the quasi-crystalline cargo characterized for β-CB by EM, and deriving from procarboxysomes [5, 59]. Variations of CcmK assembly compactness might accompany the process, something that would be in agreement with inter-hexamer distances of 9 nm measured for BMC fragment [27], as compared to 6.7 nm spacing on full BMC [26]. Alternatively, the accumulation of elastic stress during the growth of curved patches might lead to buckling transitions towards facetted icosahedral compartments, similar to those reported for viral capsids [60]. Such flattening transitions might be triggered by incorporation of other components, such as pentameric CcmL. In principle, the possibility that curved assemblies formed even in the absence of support, something theoretically feasible, was ruled out by data from two fluorescence studies that concurred to prove that formation of β-procarboxysome seeds precedes shell assembly *in vivo* [15, 16]. Nevertheless, caution is mandatory in view of the fact that these studies were based on the reconstitution of CB integrating RuBisCO and CcmK isoforms fused to bulky fluorescent protein reporters, also because other natural CcmK isoforms were still potentially expressed. Indeed, other studies indicate that hollow structures may form: round structures with purified CcmK2 from *T*. *elongatus BP-1* [51] or hollow compartments obtained after expression in plant chloroplasts of CcmK2, CcmO and CcmL from *Syn*. *elongatus PCC7942* [19]. α-CB, Pdu or Eut compartments are also known to assemble in the absence of cargo proteins [17, 61] [14].

## Conclusions

Understanding the assembly of BMC is a prerequisite for the design of future nano-reactors and molecular scaffolds, with anticipated applications in synthetic biology, nanotechnology and medicine. In this respect, structural information reported here demonstrate that CcmK1 assemble spontaneously into non-planar motifs. In contrast to its close homologue CcmK4, which formed canonical flat assemblies, CcmK1 formed curved honeycomb-like structures, whereas an intermediate situation occurred with CcmK2, this latter resulting in both flat and striped assemblies. CcmK1 behavior was ascribed to a combination of two sequence elements that are shared by CcmK isoforms from most β-cyanobacteria, pointing to a widespread property. One of these diverging structural elements localized indeed around a potential phosphate binding site that forms at the interface between assembled hexamers, providing an explanation as to why round assemblies formed solely in the presence of phosphorylated molecules. Moreover, theoretical simulations argued in favour of assembly scenarios with no preference for flat inter-hexamer geometries, and indicated that formation of flat or curved structures relied basically on the strength of adhesion to the assembly support. Overall, our data provide evidence in support of a considerable higher structural tolerance than previously imagined for BMC-H proteins, and suggest mechanisms with transitions between curved and flat assemblies that could be important for the biogenesis of carboxysomes, possibly of BMC in general.

## Materials and methods

### Cloning, expression, and protein purification

Full-length CcmK1 (UniProt entry P72760), CcmK2 (P72761), CcmK3 (P73406) and CcmK4 (P73407) genes from *Synechocystis sp*. *PCC6803* (codon-optimized for expression in *E*. *coli*) and from CcmK1 (Q03511) from *Synechococcus elongatus PCC7942* (natural sequence), the triple A^63^AN mutant and the Δ9 C-ter variant of K1^-TH^ *Syn6803*, as well as all mutants/variants of K2^-TH^ *Syn6803* were synthesized (Genecust and Twist Bioscience) with N-ter or C-ter tag extensions (showed in S1 Fig). Sequences were cloned in either a pET-15b vector using Xba1/Xho1 or Nco1/Xho1 restriction sites or in pET-26b using Nde1/Xho1 (as indicated in S1 List). Single and double amino acid exchange variants of interfacial residues were prepared from reactions combining two mutagenic primers (for K25A + R80A or for R28A + D49A), following protocols supplied with the QuikChange Lightning multisite site-directed mutagenesis kit (Agilent Technologies). The importance of these residues to interhexamer interactions was predicted by the ANCHOR theoretical algorithm (http://structure.pitt.edu/anchor). Clonal DNA was purified and verified by sequencing. Full DNA and primer sequences are provided as supplementary data (S1 List). For subsequent studies, *E*. *coli* BL21 (DE3) cells were transformed following standard protocols.

Protein synthesis was induced with 0.2 mM IPTG in LB when cells attained mid log phase (OD = 0.6–0.8) at 37°C. Expression was allowed to proceed for 3–4 hours at 37°C before cells were harvested and stored at -20°C. Frozen cells were resuspended in 1/10^th^ of culturing volume of lysis buffer (20 mM Tris-HCl, 300 mM NaCl, 10 mM imidazol, pH 7.8), supplemented with DNase I (5 μg/mL final conc) and lysozyme (0.05 mg/mL). After incubation at room temperature with gentle agitation for 10 minutes, cell were lysed at 4°C in the presence of PMSF (1 mM final conc.) by 4 cycles of 30 sec sonication at 30% power, with intermediate pauses of 1 min without sonication (VibraCell 72434, Bioblock Scientific). Insoluble material was removed by centrifugation for 20 min at 20.000 x g (4°C). Soluble fractions were loaded on cobalt-loaded TALON Superflow metal affinity resin (Clontech) conditioned at 4 to 10°C. After thoroughly washing with Sol A (20 mM NaPi, 300 mM NaCl, 10 mM imidazol, pH 7.8), elution was effected with Sol B (300 mM imidazol in Sol A). EDTA (5 mM final conc.) was added immediately after elution. Proteins were buffer-exchanged against Sol C (10 mM HEPES, 300 mM NaCl, pH 7.5) by 3–4 steps 10-fold dilution/concentration steps in Vivaspin Turbo 15, 10 kDa MWCO devices, before concentrating the protein to 1–2 mg/mL. Protein concentrations were calculated from 280 nm absorption readings, using theoretical extinction coefficients estimated from protein sequences with ExPASy ProtParam tool (http://web.expasy.org/protparam/).

### TEV-treated untagged proteins

Untagged proteins were obtained after removal of oligohistidine tags by incubation of ^HT-^K, K^-TH^ or K^-ncTK-TH^ proteins (0.5 mg/mL final conc.) in 50 mM Tris pH 8.0 / 300 mM NaCl /2 mM DTT/ 1 mM EDTA, with turbo TEV protease (GenWay, 5 μg/mL). Reactions proceeded at 25°C overnight, followed by buffer-exchange at 4°C against Sol C (exactly as described above). Proteins were concentrated to 1–2 mg/mL.

### Size-exclusion chromatography

Protein sizes were estimated by SEC using a Beckman Ultraspherogel SEC2000 column (7.5 x 300 mm) mounted on a Waters 2690 HPLC separation module. Protein solution (10–20 μL) was injected at 1 mL/min flowrate after equilibration of the column with 20 mM Tris-HCl, 300 mM NaCl at pH 7. Elution was monitored with a Waters 996 Photodiode Array Detector. Elution volumes (280 nm absorption) were used to estimate protein MW by comparison to next calibration standards run under identical conditions: Ferritin (440 kDa), Aldolase (158 kDa), Conalbumin (75 kDa), Ovalbumin (43 kDa) and Ribonuclease (13.7 kDa).

### Native-mass spectrometry

Prior to measurements, TEV-treated proteins were buffer-exchanged against 150 mM aqueous ammonium acetate at pH 8 using Amicon Ultra-0.5 mL centrifugal filters (MWCO = 10 kDa; Millipore). MS measurements were performed on a commercial electrospray ionization hybrid quadrupole time-of-flight (ESI-Q-ToF) mass spectrometer (Q-TOF Ultima API, Micromass, Manchester, U.K.) equipped with a 32k quadrupole and a high-pressure collision cell (MS Vision, Almere, Netherlands). All experiments were carried out in the positive ion mode. A commercial nano-ESI source was used at ambient temperature. Typically, 3–5 μL of sample were loaded into a Au/Pd-coated glass capillary emitter (1 μm outlet inner diameter; Thermo Scientifc, Madison, WI, USA). To generate electrospray, a voltage of 1.9–2.3 kV was applied to the emitter and a backing pressure of 0.5 bar was used to assist sample flow. In order to assure gentle transmission of the ions from atmospheric pressure to vacuum, the pressures in the first pumping compartment were increased to 3.5–4.0 mbar. The pressure inside the collision cell was set to 1*10^−2^ mbar. Several tuning parameters including the cone voltage, RF lens 1 offset, offset 1, and the collision energy offset were optimized to obtain efficient ion transfer, good signal intensity and resolution. The quadrupole transmission profile was adjusted for the desired m/z range. Mass spectra were recorded in a *m/*z 50–8000 window with a scan time of 2 s and an interscan delay of 0.1 s. Spectra were recorded using the MassLynx 4.0 software (Waters, Manchester, UK) and baseline-corrected, normalized, and smoothed using MATLAB R2015a (MathWorks, Natick, MA, USA). Fifty individual scans were typically combined to produce a mass spectrum. For calibration, CsI clusters formed by electrospraying an aqueous CsI solution (40 mg/ml) were used. The recorded spectra were averaged (50 scans), smoothed with a moving average algorithm (width of ±3 steps) and centroid spectra were generated at 80% peak height. The *m/z* axis was calibrated by fitting a polynomial function.

For tandem mass spectrometry experiments, precursor ions were isolated in the quadrupole mass analyzer and accelerated into an argon-filled linear hexapole collision cell. Various collision energy offsets were applied upstream of the collision cell, with argon at a pressure of 3.0 x 10^−2^ mbar.

Drift times for CcmK2 constructs and protein calibrants were measured by ion mobility mass spectrometry experiments performed on a hybrid quadrupole-IMS-TOF instrument (Synapt G2S HDMS, Waters, Manchester, UK). For calibration, the following standard proteins were used: concanavalin A, bovine β-lactoglobulin, bovine serum albumin, alcohol dehydrogenase, transthyretin, as described in reference [62]. Nitrogen was used as a buffer gas in the ion mobility cell operated at a nominal pressure of 1.65 mbar. Typically, ion mobility spectra were acquired for 2 minutes. The source temperature was kept at 30°C, and the pressure in the first pumping stage was increased to 3.0 mbar. Drift times of the folded calibrant protein ions were extracted and used to produce a calibration curve for collision cross section. Ω values for CcmK2 hexamers as well as for species detected at higher m/z were compared to sheet-like structures of norovirus and HBV that were determined by Heck and coworkers under the same conditions (S4 Fig) [42].

### Imaging with AFM

Proteins were 10–50 fold diluted in different solutions, adjusted to pHs 5.0 to 8.0, and containing or not commercially available additives (Sigma-Aldrich) at 0.5 mM final concentrations (as indicated in figure legends): Sol AFM1: 10 mM NaPi, 300 mM NaCl; Sol AFM2: 10 mM MES:NaOH, 300 mM NaCl; Sol AFM3: 10 mM Tris:HCl, 300 mM NaCl. Two μL of these solutions were then dispensed onto freshly-cleaved mica and proteins allowed to adsorb for 15–30 min. Samples were imaged after dilution with 150 μl of the same buffer. For conventional AFM imaging, a Multimode 8 AFM (Bruker), equipped with a 160-μm scanner (J-scanner) and oxide-sharpened Si_3_N_4_ cantilevers (k = 0.09 N·m^−1^, Olympus) was operated in contact mode in buffer at ambient temperature and pressure. Minimal loading forces of approximately 300 pN were applied during AFM imaging, at scan frequencies of 3–4 Hz using optimized feedback parameters. Images were acquired at 512 x 512 pixel resolution.

HS-AFM images were captured at between 30 and 60 Hz in buffer in AC mode using a NanoWizard ULTRA speed A (JPK) equipped with an ULTRA Speed 2.8 μm scanner and ‘Ultra-Short Cantilever’ USC-0.3 MHz probes (0.3 N·m^−1^, Nano World), or in tapping mode using a Dimension Fastscan AFM (Bruker) equipped with 30 μm Icon scanner and ScanAsyst fluid plus probes (0.7 N·m^−1^, Bruker).

Standard image analysis and treatments were initially performed using NanoScope Analysis software (Bruker). When necessary, AFM images were processed with 0 to 3^th^ order plane fitting and 0 to 3^rd^ order flattening to reduce XY tilt. HS-AFM frames were aligned using ImageJ scripts (mostly StackReg). To analyze protein dynamics from consecutive series of AFM images, images were aligned as stacks, and standard deviations evaluated using the Z-project command.

### Atomistic molecular dynamics simulations

Three hexamers were assembled using CcmK1 structure (PDB ID 3BN4) and applying symmetry operations within Swiss-PdbViewer (http://www.expasy.org/spdbv/)[63]. Interhexamer sulfates were either deleted, or replaced by phosphate, or MePi_3_ ligands, which were parameterized using the PRODRG server (http://davapc1.bioch.dundee.ac.uk/cgi-bin/prodrg)[64]. In the case of PyrR protein, the crystallized dimer was used (1AX4). After a first energy minimization within YASARA, the CcmK1 ensemble or the PyrR dimer were hydrated within a cubic cell with dimensions extending by 20 nm around protein atoms, which was filled with explicit solvent. Periodic boundary conditions were applied and the AMBER14 force field was employed. The cut-off for the Lennard-Jones potential and the short range electrostatics was 8 Ǻ. Long-range electrostatics were calculated using the Particle Mesh Ewald (PME) method with a grid spacing <0.1 nm, 4th order PME-spline, and PME tolerance of 10^−5^ for the direct space sum. Force field parameters for protein and phosphate atoms followed general AMBER force field atom type assignments. YASARA’s pKa utility was used to assign pKa values at pH 7.0. The simulation cell was neutralized with NaCl (0.9% final concentration) by iteratively placing sodium and chlorine ions at the coordinates with the lowest electrostatic potential. The entire system was energy-minimized using steepest descent minimization, in order to remove conformational stress, followed by a simulated annealing minimization until convergence (<0.05 kJ/mol/200 steps). Simulations were run at 298 K, with integration time steps for intra-molecular and inter-molecular forces of 1 fs and 2 fs, respectively. After equilibration, simulations were continued for 15 ns. This was followed by an identical second simulation, with attribution of random initial atomic velocities. Intermediate structures were saved every 250 ps.

Dihedral angle analysis and generation of figures was done with Pymol (https://www.pymol.org/).

### Coarse-grained simulations

Brownian Dynamics simulations of a coarse-grained model were implemented to analyze the assembly of CcmK hexamers. In the model, a CcmK hexamer is represented at very low resolution as a nearly spherical bead of diameter σ, described in terms of its position ***r***_***i***_ and orientation ***Ω***_***i***_. The interaction between hexamers is modeled using the same potential as in Ref. [45], which depends on three main parameters: the binding energy between hexamers, *ϵ*_0_; the preferred angle of the inter-hexamer interaction, *ν*; and the local bending stiffness dictated by *α*. The interaction between an hexamer and the substrate was modeled using a potential

$$V_{subs}(z,\Omega_{i})=\frac{5}{3}\epsilon_{s}\;\left[\frac{2}{5}{\left(\frac{\sigma_{s}}{z}\right)}^{10}-{\left(\frac{\sigma_{s}}{z}\right)}^{4}\right](1+\cos\theta_{i}),$$

where *z* is the vertical distance to the substrate, *σ*_*s*_ = *σ*/2 is the equilibrium distance, *ϵ*_*s*_ is the adhesion strength and *θ*_*i*_ is the polar angle of the orientation of hexamer i. The orientational dependence of this potential is introduced to mimic the asymmetry between the concave and convex faces of the CcmK proteins and the presence of the tags.

We worked using reduced units in terms of the diameter of hexamers *σ*, their diffusion coefficient *D*, and the binding energy *ε*_0_. The differences between CcmK proteins are modelled using different interaction parameters. In these reduced units, typical parameters used in the simulation are: binding stiffness, *α* = 0.1, preferred angle between hexamers, *ν* = 1.4 radians and torsion constant, *k*_*t*_ = 1.5. Brownian Dynamics simulations were implemented using a stochastic Euler’s algorithm with a timestep *Δt* = 10^−5^*σ*^2^/*D*. In the simulations, N hexamers are placed initially at random positions and orientations inside a cubic box with periodic boundary conditions and their dynamics is monitored for typically 4 10^8^ timesteps (corresponding to *ca*. 0.8 ms). Snapshots and movies of the simulation were prepared with VMD (http://www.ks.uiuc.edu/Research/vmd/).

## Supporting information

S1 Table Molecular weight determination of CcmK constructs from native ESI-MS spectra. Experimental masses for neutral species were calculated from m/z values of multiply- charged ions attributed to monomers and hexamers in Fig 1 and S3 Fig. Standard deviations are shown in parenthesis. These values are compared to theoretical molecular weights calculated from amino acid sequences. Deviations between experimental and theoretical values are indicated. Proteins tagged at C-terminus result in experimental MW that differ from theoretical values by a mass that is compatible with loss of the first methionine residue (131.04 Da). With both *Syn6803* CcmK2 constructs, potential sheet-like preassemblies of high MW did not always match to integer combinations of hexameric subunits.
(DOCX)

S1 List Full DNA sequences for expression of studied CcmK variants, and primers for mutagenesis work. (DOCX)

S1 Fig Schematic representation of CcmK constructs. Boxes are to schematize emplacement of wild-type protein sequences, which are indicated on top for each isoform. Only starting and ending stretches are indicated within each box. N-ter or C-ter oligohistidine tags are indicated in red, in green TEV cleavable sequences. TEV-proteolyzed products are not indicated for proteins that could not be purified.
(TIF)

S2 Fig Expression and solubility of engineered CcmK constructs. Coomassie-stained SDS-PAGE showing the presence or absence of bands corresponding to over-expressed proteins in total cell content (C) or fractions remaining soluble after lysis and centrifugation (S). Panel *A*: N-ter His_4_-tagged CcmK isoforms (^HT-^K); *B*: C-ter His_4_-tagged CcmK isoforms (K^-TH,^, *B*); *C*: untagged proteins (^Un^K). Proteins of interest have theoretical MW comprised between 12.8 and 14.1 kDa (region indicated with dashed box). Amounts equivalent to approximately 4 μL at OD^280nm^ = 5 were loaded in each lane.
(TIF)

S3 Fig Oligomerization and potential assembling of CcmK1-4 revealed by n-MS. Positive-ion mode native ESI-MS spectra from CcmK isoforms: *A*, *Syn6803* K2^-^; *B*, *Syn6803* K4^-^*; C*, *Syn7942* K2^-H^. For panels A and B, tags were removed by TEV protease treatments prior to spraying. Data support the occurrence of hexamers in solution. In addition, potential assembling intermediates with higher oligomerization state (see main text) were noticed in experiments with K2^-^ 6803 isoforms (panel *A*). Species of similar, but not identical MW are detected for K4^-TH^ (*B*), pointing to sample proteolytic degradation (portion enlarged in the inset, for clarity). Right panels present collisional activation data collected on selected hexamer precursor ions (asterisk). An asymmetric charge partitioning is noticed, hexamers dissociating into highly-charged monomer and pentamer species carrying the remaining charge. Species m/z values and charges are indicated above most intense peaks. Molecular weights of neutral species obtained by convolution of these data are compiled in S1 Table.
(TIF)

S4 Fig Potential assembly intermediates of *Syn6803* CcmK2 revealed by native IMS-MS. Collisional cross sections (CCS) were determined for selected species detected in Fig 1B with TEV-treated ^-^K2 (red triangles) or in S3B Fig for K2^-^ (blue triangles). CCS data for hexameric species (approx. MW 70 kDa) were measured on species with m/z 3799 for ^-^K2 and m/z 4522 for K2^-^, whereas CCS values for higher MW species were estimated from peaks at m/z 4999 for ^-^K2 and m/z 5563 for K2^-^. Experimental Ω values reported before for sheet-like partially disassembled viruses (empty squares) and globular proteins (open circles)^38^, as well as globular proteins measured here (blue filled circles) are plotted for comparison.
(TIF)

S5 Fig Phosphates and sulfonates promote supra-hexagonal organization of CcmK1 from *Syn sp*. PCC6803. AFM images were recorded after absorption on mica of 100 ng of K1^-TH^ 6803 conditioned in saline Tris buffer pH7.0 including next additives: *A*, nothing; *B*, 0.5mM ADP; *C*, 0.5 mM GtetraPi; *D*, 0.5 mM NaHCO_3_; *E*, 1 mM HEPES; *F*, 0.5 mM 3-phosphoglyceric acid; *G*, 0.5 mM Na_2_SO_4_; *H* 0.5 mM ribulose-1,5-biphosphate; *I*, 0.5 mM MgCl_2_. Images are 1 μm large, 2 μm for panel C.
(TIF)

S6 Fig Dynamics of formation of polygonal mosaics with K1^-TH^ 6803 revealed by HS-AFM. Shown are sixteen non-aligned time-lapse AFM images selected from a 1hr long HS-AFM movie recorded at 4 sec per frame (S1 Movie). White circles are depicted to indicate the emplacement of one of the earliest assembly events that lead to a curved honeycomb-like patch. Images were captured after injection of 50 μL of K1^-TH^ 6803 (40 μg/mL) in 10 mM NaPi/300 mM NaCl at pH 6.5 once the cantilever immersed probe engaged above the mica in 50 μL of the same solution.
(TIF)

S7 Fig Dynamic stability of polygonal mosaics with K1^-TH^ 6803 revealed by HS-AFM. *Left*, single frame representative of assembled motifs monitored over 20 min at 10 sec intervals (S2 Movie). Prior to imaging, the protein was allowed to assemble for 30 min on mica in the presence of 10 mM MES, 300 mM NaCl, pH 7. *Centre*, average image obtained after alignment of 123 recorded frames. *Right*, representation of standard deviation between images, with grey scale ranging from white to black for highest difference to no change, respectively.
(TIF)

S8 Fig Expression and solubility of single- and double-point mutants of K1^-TH^ 6803. Coomassie-stained SDS-PAGE showing the presence or absence of bands corresponding to over-produced proteins (theoretical MW of 14 kDa) in total cell content (C) or fractions remaining soluble after lysis and centrifugation (S). Indicated with arrows are mutants that could not be purified. Total amount loaded in each lane is equivalent to approximately 4 μL of a protein solution at OD^280nm^ = 5.
(TIF)

S9 Fig Mutation of interfacial residues abrogate formation of supra-hexagonal arrangements of CcmK1 from *Syn sp*. PCC6803. AFM images were recorded after absorption on mica of 100 ng of K1^-TH^ 6803 indicated mutants conditioned in saline MES buffers at pH 6.0: R28A (panel *A)*, D49A (*B*), R80A (*C*), and double mutants R28A/R80A (*D*) or D49A/R80A at pH 6.5 (*E*). Only shown one image from several performed for each mutant after deposition at pH 6.0, 6.5 and 7.0. Image sizes are 5 times those of indicated scale bars.
(TIF)

S10 Fig Sequence and structural comparison of K1 6803 with K2 6803 and K2 7942. *A*, Sequence alignments highlighting positions differing among the three proteins. Two major regions acumulate most of differences: the C-ter side of α2 helix (green bar) and the C-ter extension (blue bar) present only in K1 6803 (also in K4 6803, not shown). Secondary structural elements were extracted from K1 6803 structure (PDB ID 3BN4) are indicated on top of the alignment. *B*, Structural differences observed for C-ter side in helix α2 of K1 6803 (3BN4, in green) as compared to K2 6803 (2A1B, dark grey) or K2 7942 (4OX7, light grey). Side-chain atoms of some selected residues are represented as sticks: carbon with same colors as cartoon, nitrogens in blue and oxygens red. A neighboring hexamer generated with symmetry operations on 3BN4 structure is shown on the bottom right side (black cartoon/sticks). Modeled sulfate is represented with sulfur and oxygen atoms as yellow and red sticks, respectively.
(TIF)

S11 Fig Sequence alignment of α2 helical residues of BMC-H from β-cyanobacteria. Only shown sequence stretch around residues corresponding to amino acids 63–66 of CccmK1 from Syn Sp. PCC6803. Sequence names: NCBI entry code_species name_Rub/Sat, where Rub or Sat indicate whether the corresponding ORF lies at the same loci as RuBisCO subunits or in a satellite loci [as defined in reference [1]]. Green frames highlight sequences that presumably might lead to similar structural consequences as NI 63–64 residues of *Syn6803* CcmK1.
(TIF)

S12 Fig Sequence alignment of C-terminal residues of BMC-H from β-cyanobacteria. Only shown sequence stretch around residues corresponding to the C-terminal extension. Other details as for S11 Fig.
(TIF)

S1 Movie Formation of polygonal mosaics with K1^-TH^ 6803 revealed by HS-AFM. Data recorded at 4 sec per frame. Snapshots presented in S6 Fig.
(AVI)

S2 Movie Dynamic fluctuations of polygonal mosaics of K1^-TH^ 6803 revealed by HS-AFM. Data recorded at 10 sec per frame. Same data are presented in S7 Fig.
(AVI)

S3 Movie Molecular dynamics of CcmK1 in complex with MePi_3_ ligand at the potential phosphate binding funnel. The movie was mounted from snapshots taken at 0.25 ns intervals from an all-atom MD simulations (15 ns) on an ensemble of three CcmK1 *Syn6803* hexamers, originally positioned as in the published crystal structure (PDB code 3BN4), with MePi_3_ replacing interhexamer crystallographic sulfates. A cartoon representation is drawn, with each hexamer colored differently (excluding). For clarity, snapshot structures were superimposed taking only in consideration coordinates from main-chain atoms of chain A (in black). Water and salt molecules were excluded from the representation. Torsion tilting and bending angles measured throughout this simulation and a similar independent second MD run (with random seed attribution of initial velocities) are presented in Fig 5B.
(AVI)

S4 Movie Molecular dynamics of CcmK1 in complex with MePi_3_ ligand at the potential phosphate binding funnel. This movie shows a close-up view of S3 Movie, centered on the potential phosphate binding funnel where MePi_3_ is bound. Hexamers A and B are shown as grey and blue cartoons, respectively. The third hexamer is omitted for clarity. The ligand and the side-chains of main residues of the ligand-binding funnel are shown as sticks. Carbon, nitrogen, oxygen and phosphates, are colored green, blue, red and orange, respectively. Dashed-lined display the evolution of polar interactions established between the ligand and main surrounding residues (R66, K25 and R80) throughout the course of the MD run.
(AVI)

S5 Movie Molecular dynamics of CcmK1 in the absence of interfacial ligand. All-atom MD were run on a tri-hexamer CcmK1 ensemble, under same conditions as for data presented in S3 and S4 Movies, with exception that no ligand was included at the emplacement of crystallographic sulfates. Torsion tilting and bending angles measured throughout this simulation and a similar independent second MD run (with random seed attribution of initial velocities) are presented in Fig 5B.
(AVI)

S6 Movie Molecular dynamics of *B*. *subtilis* PyrR dimer. All-atom MD of *B*. *subtilis* pyrimidine biosynthesis operon repressor (PDB code 1A4X). Simulations were run exactly as for S3 Movie, but including two interacting units (unlike with 3 hexamers in previous MDs with CcmK1). The movie was prepared exactly as for S3 Movie. Torsion tilting and bending angles measured throughout this simulation and a similar independent second MD run (with random seed attribution of initial velocities) are presented in Fig 5B.
(AVI)

S7 Movie CcmK assembly dynamics simulated with a coarse-grained model. The simulation shows the kinetics of formation on the substrate of stable patches of 15–20 hexagons with raised borders that resemble structures formed during the assembly of CcmK1. The movie was generated with VMD from snapshots taken every 10^5^ timesteps of a coarse-grained simulation run with the same parameters as in Fig 5D (i. ε _0_ = 8.3 *k*_*B*_*T*, *n* = 1.4 rad, ε_ads_ = 2.5 *k*_*B*_*T*, *α* = 0.1 and hexamer concentration 0.0125 *σ*^*-3*^). In the simulation, 800 hexamers were used and they were initially placed randomly on the bulk of the solution. The plane of the solid support appears blue and only hexamers near the surface are plotted. The hexamers are colored according to their height over the surface in a color scale ranging from white (for hexamers adsorbed on the surface) to red (for hexamers at a distance σ away from the substrate).
(MPG)
