# Supplementary material for: Spontaneous non-canonical assembly of CcmK hexameric components from β-carboxysome shells of cyanobacteria
Source: PLoS One. 2017 Sep 21;12(9):e0185109. doi: 10.1371/journal.pone.0185109 (PMC5608322; doi:10.1371/journal.pone.0185109)
Supplement: S1 Table — Experimental masses for neutral species were calculated from m/z values of multiply- charged ions attributed to monomers and hexamers in Fig 1 and S3 Fig. Standard deviations are shown in parenthesis. These values are compared to theoretical molecular weights calculated from amino acid sequences. Deviations between experimental and theoretical values are indicated. Proteins tagged at C-terminus result in experimental MW that differ from theoretical values by a mass that is compatible with loss of the first methionine residue (131.04 Da). With both Syn6803 CcmK2 constructs, potential sheet-like preassemblies of high MW did not always match to integer combinations of hexameric subunits. (DOCX) [file pone.0185109.s001.docx]

**Table S1 – Molecular weight determination of CcmK constructs from native ESI-MS spectra.**

|  | Theoretical  MW | Experimental MW | Mass deviation^a^ | Deviation  from N-mer^b^ | N |
| --- | --- | --- | --- | --- | --- |
| *Syn6803* K1^-^ | 13153.0 | 13021.5 (0.2) | - 131.5 | - | 1 |
|  | 78918.0 | 78237 (23) | - 687 | + 108 | 6 |
| *Syn6803* K2^-^ | 12185.8 | 12054.6 (0.4) | -131.2 | - | 1 |
|  | 73114.8 | 72359 (30) | -754.8 | +31 | 6 |
|  | 341202.4 | 345256 (105) | +4057.6 | +7731 | 28 |
|  | 353388.2 | 355293 (111) | +1911.8 | +5717 | 29 |
|  | 365574.0 | 367265 (108) | 1726.0 | +5662 | 30 |
| *Syn6803* ^-^K2 | 11317.9 | 11328 (3) | + 9.8 | - | 1 |
|  | 67907.4 | 67973 (29) | + 62.6 | +8 | 6 |
|  | 237675.9 | 239267 (122) | +1624.1 | +1370 | 21 |
|  | 260311.7 | 263261 (115) | +29488.3 | +2710 | 23 |
|  | 271629.6 | 275768 (107) | +4170.4 | +3880 | 24 |
| *Syn6803* K4^-^ | 12953.8 | 12822.6 (0.4) | - 131.2 | - | 1 |
|  | 77722.8 | 77594 (65) | - 132.8 | + 654 | 6 |
| *Syn6803* ^-^K4 | 12085.8 | 12091 (7) | + 5.1 | - | 1 |
|  | 72514.8 | 72598 (16) | + 85.2 | + 60 | 6 |
| *Syn7942* K2^-H^ | 12182.0 | 12050.6 (0.3) | - 131.4 | - | 1 |
|  | 73092.0 | 72368 (22) | - 722.0 | + 66 | 6 |

^a^ Deviation with regard to theoretical molecular weight; ^b^ Deviation from molecular weight of species composed by N times the experimental monomer mass.

Experimental masses for neutral species were calculated from m/z values of multiply-charged ions attributed to monomers and hexamers in figures 1 and S3. Standard deviations are indicated within parenthesis. These values are compared to theoretical molecular weights calculated from amino acid sequences. Deviations between experimental and theoretical values are indicated. Proteins tagged at C-terminus result in experimental MW that differ from theoretical values by a mass that is compatible with loss of the first methionine residue (131.04 Da). With both *Syn6803* CcmK2 constructs, potential sheet-like preassemblies of high MW did not always match to integer combinations of hexameric subunits.
