# Supplementary material for: Spontaneous non-canonical assembly of CcmK hexameric components from β-carboxysome shells of cyanobacteria
Source: PLoS One. 2017 Sep 21;12(9):e0185109. doi: 10.1371/journal.pone.0185109 (PMC5608322; doi:10.1371/journal.pone.0185109)
Supplement: S1 List — (DOCX) [file pone.0185109.s002.docx]

Underlined: Xba1 (or NCO1) and Xho1 sites for transfer to PET15b

nde1 and xho1 for transfer to pet26b

IN BOLD characters : start and stop translation sites

In blue: beginning of CcmK sequence

In red: mutated codons

> ^HT-^K1 6803

cccctctagAAATAAGATTTAAATACTTTAAGAAGGAGATATACC**ATG**GCACATCACCATCATGctagCGGCGAAAATCTGTACTTCCAGGGTGCC**ATG**GCAATCGCTGTAGGTATGATCGAAACTCTGGGGTTTCCGGCTGTTGTGGAAGCAGCCGATAGCATGGTAAAAGCGGCGCGCGTGACCTTAGTGGGCTATGAAAAGATTGGCAGCGGTCGTGTCACCGTTATTGTTCGCGGGGATGTCAGCGAGGTGCAAGCGTCAGTGACGGCGGGTATCGAAAATATCCGTCGTGTAAACGGTGGAGAAGTACTGTCAAACCATATCATCGCACGCCCACATGAAAATCTGGAGTATGTTTTACCGATTCGCTATACGGAAGCTGTGGAGCAGTTTCGTGAGATTGTAAACCCAAGCATCATCCGCCGTGCT**TAA**GATCCACTTCtcgagGAT

> K1^-TH^ 6803

cccctctagAAATAAGATTTAAATACTTTAAGAAGGAGATATACc**atg**GCAATCGCTGTAGGTATGATCGAAACTCTGGGGTTTCCGGCTGTTGTGGAAGCAGCCGATAGCATGGTAAAAGCGGCGCGCGTGACCTTAGTGGGCTATGAAAAGATTGGCAGCGGTCGTGTCACCGTTATTGTTCGCGGGGATGTCAGCGAGGTGCAAGCGTCAGTGACGGCGGGTATCGAAAATATCCGTCGTGTAAACGGTGGAGAAGTACTGTCAAACCATATCATCGCACGCCCACATGAAAATCTGGAGTATGTTTTACCGATTCGCTATACGGAAGCTGTGGAGCAGTTTCGTGAGATTGTAAACCCAAGCATCATCCGCCGTGCGGGTAGCGGCGAAAATCTGTACTTCCAGAGTGCggccGCACATCACCATCAT**TGATAG**GATCCACTTCtcgaggat

> ^Un^K1 6803

cccctctagAAATAAGATTTAAATACTTTAAGAAGGAGATATACc**atg**GCAATCGCTGTAGGTATGATCGAAACTCTGGGGTTTCCGGCTGTTGTGGAAGCAGCCGATAGCATGGTAAAAGCGGCGCGCGTGACCTTAGTGGGCTATGAAAAGATTGGCAGCGGTCGTGTCACCGTTATTGTTCGCGGGGATGTCAGCGAGGTGCAAGCGTCAGTGACGGCGGGTATCGAAAATATCCGTCGTGTAAACGGTGGAGAAGTACTGTCAAACCATATCATCGCACGCCCACATGAAAATCTGGAGTATGTTTTACCGATTCGCTATACGGAAGCTGTGGAGCAGTTTCGTGAGATTGTAAACCCAAGCATCATCCGCCGTGCT**TAA**GATCCACTTCtcgagGAT

> K1^-H^ 6803

cccctctagaaataattttgtttaactttaagaaggagatatacat**aTG**TCAATCGCTGTAGGTATGATCGAAACTCTGGGGTTTCCGGCTGTTGTGGAAGCAGCCGATAGCATGGTAAAAGCGGCGCGCGTGACCTTAGTGGGCTATGAAAAGATTGGCAGCGGTCGTGTCACCGTTATTGTTCGCGGGGATGTCAGCGAGGTGCAAGCGTCAGTGACGGCGGGTATCGAAAATATCCGACGTGTAAACGGTGGAGAAGTACTGTCAAACCATATCATCGCACGCCCACATGAAAATCTGGAGTATGTTTTACCGATTCGGTATACGGAAGCTGTGGAGCAGTTTCGTGAGATTGTAAACCCAAGCATCATCCGGCGTGCGGCGGCCCTTGAACACCATCACCATCACCAT**TGATAA**Ccatggatatcggaattaattcggatccgaattcgagctccgtcgacaagcttgcggccgcactcgagcac

> K1^-ncTK-TH^ 6803

cccctctagaaataattttgtttaactttaagaaggagatatacc**atg**GCAATCGCTGTAGGTATGATCGAAACTCTGGG

GTTTCCGGCTGTTGTGGAAGCAGCCGATAGCATGGTAAAAGCGGCGCGCGTGACCTTAGTGGGCTATGAAAAGATTGGCA

GCGGTCGTGTCACCGTTATTGTTCGCGGGGATGTCAGCGAGGTGCAAGCGTCAGTGACGGCGGGTATCGAAAATATCCGT

CGTGTAAACGGTGGAGAAGTACTGTCAAACCATATCATCGCACGCCCACATGAAAATCTGGAGTATGTTTTACCGATTCG

CTATACGGAAGCTGTGGAGCAGTTTCGTGAGATTGTAAACCCAAGCATCATCCGCCGTGCGGGTAGCGGCGAAAATCTGT

ACTTCGCAAGTGCggccGCAAAAAAGAAAGGCGGTAGCGGCGAAAATCTGTACTTCCAGAGTGCggccGCACATCACCAT

CATTGATAGGATCCACTTCtcgaggat

> AAN K1^-TH^ 6803

agatatacat**atg**GCAATCGCTGTAGGTATGATCGAAACTCTGGGGTTTCCGGCTGTTGTGGAAGCAGCCGATAGCATGG

TAAAAGCGGCGCGCGTGACCTTAGTGGGCTATGAAAAGATTGGCAGCGGTCGTGTCACCGTTATTGTTCGCGGGGATGTC

AGCGAGGTGCAAGCGTCAGTGACGGCGGGTATCGAAGCCGCGAATCGTGTAAACGGTGGAGAAGTACTGTCAAACCATAT

CATCGCACGCCCACATGAAAATCTGGAGTATGTTTTACCGATTCGCTATACGGAAGCTGTGGAGCAGTTTCGTGAGATTG

TAAACCCAAGCATCATCCGCCGTGCGGGTAGCGGCGAAAATCTGTACTTCCAGAGTGCggccGCACATCACCATCATTGA

TAGctcgagcaccacca

> Δ9 K1^-TH^ 6803

agatatacat**atg**GCAATCGCTGTAGGTATGATCGAAACTCTGGGGTTTCCGGCTGTTGTGGAAGCAGCCGATAGCATGG

TAAAAGCGGCGCGCGTGACCTTAGTGGGCTATGAAAAGATTGGCAGCGGTCGTGTCACCGTTATTGTTCGCGGGGATGTC

AGCGAGGTGCAAGCGTCAGTGACGGCGGGTATCGAAAATATCCGTCGTGTAAACGGTGGAGAAGTACTGTCAAACCATAT

CATCGCACGCCCACATGAAAATCTGGAGTATGTTTTACCGATTCGCTATACGGAAGCTGTGGAGCAGTTTCGTGAGGCGG

GTAGCGGCGAAAATCTGTACTTCCAGAGTGCggccGCACATCACCATCATTGATAGctcgagcaccacca

> ^HT-^K2 6803

cccctctagAAATAAGATTTAAATACTTTAAGAAGGAGATATACC**ATG**GCACATCACCATCATGctagCGGCGAAAATCTGTACTTCCAGGGTGCC**ATG**GCAATCGCTGTGGGTATGATCGAAACACGCGGGTTTCCAGCGGTTGTGGAGGCGGCGGATTCAATGGTAAAAGCAGCGCGCGTTACCTTAGTGGGCTATGAAAAGATTGGCAGCGGTCGTGTAACCGTTATTGTGCGTGGGGATGTTAGCGAAGTCCAGGCAAGCGTCAGCGCCGGCATCGAGGCGGCAAATCGTGTGAATGGTGGGGAAGTACTGTCAACGCATATCATCGCACGCCCACATGAAAATCTGGAGTATGTTTTACCGATCCGTTATACCGAAGAAGTTGAACAGTTCCGTACGTACGCT**TAA**GATCCACTTCtcgaggat

> K2^-TH^ 6803

cccctctagAAATAAGATTTAAATACTTTAAGAAGGAGATATACc**atg**GCAATCGCTGTGGGTATGATCGAAACACGCGGGTTTCCAGCGGTTGTGGAGGCGGCGGATTCAATGGTAAAAGCAGCGCGCGTTACCTTAGTGGGCTATGAAAAGATTGGCAGCGGTCGTGTAACCGTTATTGTGCGTGGGGATGTTAGCGAAGTCCAGGCAAGCGTCAGCGCCGGCATCGAGGCGGCAAATCGTGTGAATGGTGGGGAAGTACTGTCAACGCATATCATCGCACGCCCACATGAAAATCTGGAGTATGTTTTACCGATCCGTTATACCGAAGAAGTTGAACAGTTCCGTACGTACGCGGGTAGCGGCGAAAATCTGTACTTCCAGAGTGCggccGCACATCACCATCAT**TGATAG**GATCCACTTCtcgaggat

> ^Un^K2 6803

cccctctagAAATAAGATTTAAATACTTTAAGAAGGAGATATACc**atg**GCAATCGCTGTGGGTATGATCGAAACACGCGGGTTTCCAGCGGTTGTGGAGGCGGCGGATTCAATGGTAAAAGCAGCGCGCGTTACCTTAGTGGGCTATGAAAAGATTGGCAGCGGTCGTGTAACCGTTATTGTGCGTGGGGATGTTAGCGAAGTCCAGGCAAGCGTCAGCGCCGGCATCGAGGCGGCAAATCGTGTGAATGGTGGGGAAGTACTGTCAACGCATATCATCGCACGCCCACATGAAAATCTGGAGTATGTTTTACCGATCCGTTATACCGAAGAAGTTGAACAGTTCCGTACGTACGCT**TAA**GATCCACTTCtcgaggat

> NIR K2^-TH^ 6803

agatatacc**atg**GCAATCGCTGTGGGTATGATCGAAACACGCGGGTTTCCAGCGGTTGTGGAGGCGGCGGATTCAATGGT

AAAAGCAGCGCGCGTTACCTTAGTGGGCTATGAAAAGATTGGCAGCGGTCGTGTAACCGTTATTGTGCGTGGGGATGTTA

GCGAAGTCCAGGCAAGCGTCAGCGCCGGCATCGAGAATATCCGTCGTGTGAATGGTGGGGAAGTACTGTCAACGCATATC

ATCGCACGCCCACATGAAAATCTGGAGTATGTTTTACCGATCCGTTATACCGAAGAAGTTGAACAGTTCCGTACGTACGC

GGGTAGCGGCGAAAATCTGTACTTCCAGAGTGCggccGCACATCACCATCATTGATAGGATCCACTTCtcgaggat

> 9Cter K2^-TH^ 6803

agatatacc**atg**GCAATCGCTGTGGGTATGATCGAAACACGCGGGTTTCCAGCGGTTGTGGAGGCGGCGGATTCAATGGT

AAAAGCAGCGCGCGTTACCTTAGTGGGCTATGAAAAGATTGGCAGCGGTCGTGTAACCGTTATTGTGCGTGGGGATGTTA

GCGAAGTCCAGGCAAGCGTCAGCGCCGGCATCGAGGCGGCAAATCGTGTGAATGGTGGGGAAGTACTGTCAACGCATATC

ATCGCACGCCCACATGAAAATCTGGAGTATGTTTTACCGATCCGTTATACCGAAGAAGTTGAACAGTTCCGTGAGATTGT

AAACCCAAGCATCATCCGCCGTGCGGGTAGCGGCGAAAATCTGTACTTCCAGAGTGCggccGCACATCACCATCATTGAT

AGGATCCACTTCtcgaggat

> NIR/9Cter K2^-TH^ 6803

agatatacc**atg**GCAATCGCTGTGGGTATGATCGAAACACGCGGGTTTCCAGCGGTTGTGGAGGCGGCGGATTCAATGGT

AAAAGCAGCGCGCGTTACCTTAGTGGGCTATGAAAAGATTGGCAGCGGTCGTGTAACCGTTATTGTGCGTGGGGATGTTA

GCGAAGTCCAGGCAAGCGTCAGCGCCGGCATCGAGAATATCCGTCGTGTGAATGGTGGGGAAGTACTGTCAACGCATATC

ATCGCACGCCCACATGAAAATCTGGAGTATGTTTTACCGATCCGTTATACCGAAGAAGTTGAACAGTTCCGTGAGATTGT

AAACCCAAGCATCATCCGCCGTGCGGGTAGCGGCGAAAATCTGTACTTCCAGAGTGCggccGCACATCACCATCATTGAT

AGGATCCACTTCtcgaggat

> ^HT-^K3 6803

cccctctagAAATAAGATTTAAATACTTTAAGAAGGAGATATACC**ATG**GCACATCACCATCATGctagCGGCGAAAATCTGTACTTCCAGGGTGCC**ATG**GCACAAGCGGTGGGAGTGATTCAAACCTTGGGCTTTCCGAGCGTGTTAGCGGCGGCGGATGCGATGCTAAAAGGGGGCCGGGTGACGCTGGTGTATTATGACCTGGCTGAACGAGGCAACTTTGTAGTAGCAATCCGAGGTCCCGTATCAGAGGTTAACCTTTCGATGAAGATGGGATTAGCAGCGGTAAACGAGTCCGTCATGGGAGGTGAAATCGTTAGCCATTATATTGTGCCGAACCCGCCCGAAAATGTGCTGGCGGTTCTGCCAGTGGAGTATACCGAAAAGGTTGCTCGTTTCCGGACT**TAA**GATCCACTTCtcgaggatttttttg

> K3^-TH^ 6803

CccctctagAAATAAGATTTAAATACTTTAAGAAGGAGATATACc**atg**GCACAAGCGGTGGGAGTGATTCAAACCTTGGGCTTTCCGAGCGTGTTAGCGGCGGCGGATGCGATGCTAAAAGGGGGCCGGGTGACGCTGGTGTATTATGACCTGGCTGAACGAGGCAACTTTGTAGTAGCAATCCGAGGTCCCGTATCAGAGGTTAACCTTTCGATGAAGATGGGATTAGCAGCGGTAAACGAGTCCGTCATGGGAGGTGAAATCGTTAGCCATTATATTGTGCCGAACCCGCCCGAAAATGTGCTGGCGGTTCTGCCAGTGGAGTATACCGAAAAGGTTGCTCGTTTCCGGACGGGTAGCGGCGAAAATCTGTACTTCCAGAGTGCggccGCACATCACCATCAT**TGATAG**GATCCACTTCtcgaggat

> ^Un^K3 6803

CccctctagAAATAAGATTTAAATACTTTAAGAAGGAGATATACc**atg**GCACAAGCGGTGGGAGTGATTCAAACCTTGGGCTTTCCGAGCGTGTTAGCGGCGGCGGATGCGATGCTAAAAGGGGGCCGGGTGACGCTGGTGTATTATGACCTGGCTGAACGAGGCAACTTTGTAGTAGCAATCCGAGGTCCCGTATCAGAGGTTAACCTTTCGATGAAGATGGGATTAGCAGCGGTAAACGAGTCCGTCATGGGAGGTGAAATCGTTAGCCATTATATTGTGCCGAACCCGCCCGAAAATGTGCTGGCGGTTCTGCCAGTGGAGTATACCGAAAAGGTTGCTCGTTTCCGGACT**TAA**GATCCACTTCtcgaggatttttttg

> ^HT-^K4 6803

cccctctagAAATAAGATTTAAATACTTTAAGAAGGAGATATACC**ATG**GCACATCACCATCATGctagCGGCGAAAATCTGTACTTCCAGGGTGCC**ATG**GCAGCCCAGAGCGCCGTGGGCAGCATTGAAACCATTGGCTTTCCGGGCATTCTTGCCGCCGCGGATGCGATGGTAAAAGCTGGTCGCATTACCATTGTGGGCTATATTCGTGCGGGCTCTGCGCGCTTTACGCTGAACATTCGTGGGGATGTGCAGGAAGTTAAAACGGCGATGGCTGCGGGCATCGATGCCATCAACCGTACAGAAGGAGCCGATGTGAAAACCTGGGTCATTATTCCGCGCCCACATGAAAATGTCGTTGCGGTTCTGCCGATCGATTTTAGCCCTGAAGTAGAACCCTTTCGCGAAGCAGCGGAGGGCCTGAACCGTCGCGCT**TAA**GATCCACTTCtcgaggat

> K4^-TH^ 6803

cccctctagAAATAAGATTTAAATACTTTAAGAAGGAGATATACc**atg**GCAGCCCAGAGCGCCGTGGGCAGCATTGAAACCATTGGCTTTCCGGGCATTCTTGCCGCCGCGGATGCGATGGTAAAAGCTGGTCGCATTACCATTGTGGGCTATATTCGTGCGGGCTCTGCGCGCTTTACGCTGAACATTCGTGGGGATGTGCAGGAAGTTAAAACGGCGATGGCTGCGGGCATCGATGCCATCAACCGTACAGAAGGAGCCGATGTGAAAACCTGGGTCATTATTCCGCGCCCACATGAAAATGTCGTTGCGGTTCTGCCGATCGATTTTAGCCCTGAAGTAGAACCCTTTCGCGAAGCAGCGGAGGGCCTGAACCGTCGCGCGGGTAGCGGCGAAAATCTGTACTTCCAGAGTGCggccGCACATCACCATCAT**TGATAG**GATCCACTTCtcgaggat

> ^Un^K4 6803

cccctctagAAATAAGATTTAAATACTTTAAGAAGGAGATATACc**atg**GCAGCCCAGAGCGCCGTGGGCAGCATTGAAACCATTGGCTTTCCGGGCATTCTTGCCGCCGCGGATGCGATGGTAAAAGCTGGTCGCATTACCATTGTGGGCTATATTCGTGCGGGCTCTGCGCGCTTTACGCTGAACATTCGTGGGGATGTGCAGGAAGTTAAAACGGCGATGGCTGCGGGCATCGATGCCATCAACCGTACAGAAGGAGCCGATGTGAAAACCTGGGTCATTATTCCGCGCCCACATGAAAATGTCGTTGCGGTTCTGCCGATCGATTTTAGCCCTGAAGTAGAACCCTTTCGCGAAGCAGCGGAGGGCCTGAACCGTCGCGCT**TAA**GATCCACTTCtcgaggat

> ^H-^K2 7942

cccctctagaaataattttgtttaactttaagaaggagatatacat**aTG**GCACATCACCATCATCCGGGTGGCGGCAGCGGT

**ATG**CCGATTGCGGTGGGGATGATTGAGACGCTCGGGTTCCCTGCGGTCGTGGAAGCGGCGGACGCGATGGTGAAGGCGGCGCGGGTGACGCTCGTGGGGTACGAGAAGATTGGGTCGGGGCGGGTGACGGTGATTGTGCGTGGAGATGTCTCTGAAGTCCAAGCTTCTGTTTCGGCGGGGCTCGACTCGGCGAAGCGGGTGGCGGGAGGTGAAGTGCTCTCGCACCACATTATTGCCCGTCCTCACGAAAACTTAGAATACGTCCTGCCTATTAGGTATACTGAAGCTGTTGAACAATTTAGAATG**TAATGA**Ctcgagcac

> K2^-H^ 7942

Cccctctagaaataattttgtttaactttaagaaggagatatacc**aTG**CCGATTGCGGTGGGGATGATTGAGACGCTCGGGTTCCCTGCGGTCGTGGAAGCGGCGGACGCGATGGTGAAGGCGGCGCGGGTGACGCTCGTGGGGTACGAGAAGATTGGGTCGGGGCGGGTGACGGTGATTGTGCGTGGAGATGTCTCTGAAGTCCAAGCTTCTGTTTCGGCGGGGCTCGACTCGGCGAAGCGGGTGGCGGGAGGTGAAGTGCTCTCGCACCACATTATTGCCCGTCCTCACGAAAACTTAGAATACGTCCTGCCTATTAGGTATACTGAAGCTGTTGAACAATTTAGAATGGCggccgcacttgagcaccaccaccaccaccac**tga**GATCCACTTCtcgaggat

**PRIMERS:**

| MuTANT | Sequence |
| --- | --- |
| K25A | GCAGCCGATAGCATGGTAGCAGCGGCGCGCGTG |
| R28A | TAGCATGGTAAAAGCGGCGGCAGTGACCTTAGTGGGCTATGAAAAG |
| D49A | CGTTATTGTTCGCGGGGCTGTCAGCGAGGTGCAAG |
| R80A | CTGTCAAACCATATCATCGCAGCCCCACATGAAAATCTGGAGTATG |
