# Supplementary figures and images for: Spontaneous non-canonical assembly of CcmK hexameric components from β-carboxysome shells of cyanobacteria
Source: PLoS One. 2017 Sep 21;12(9):e0185109. doi: 10.1371/journal.pone.0185109 (PMC5608322; doi:10.1371/journal.pone.0185109)

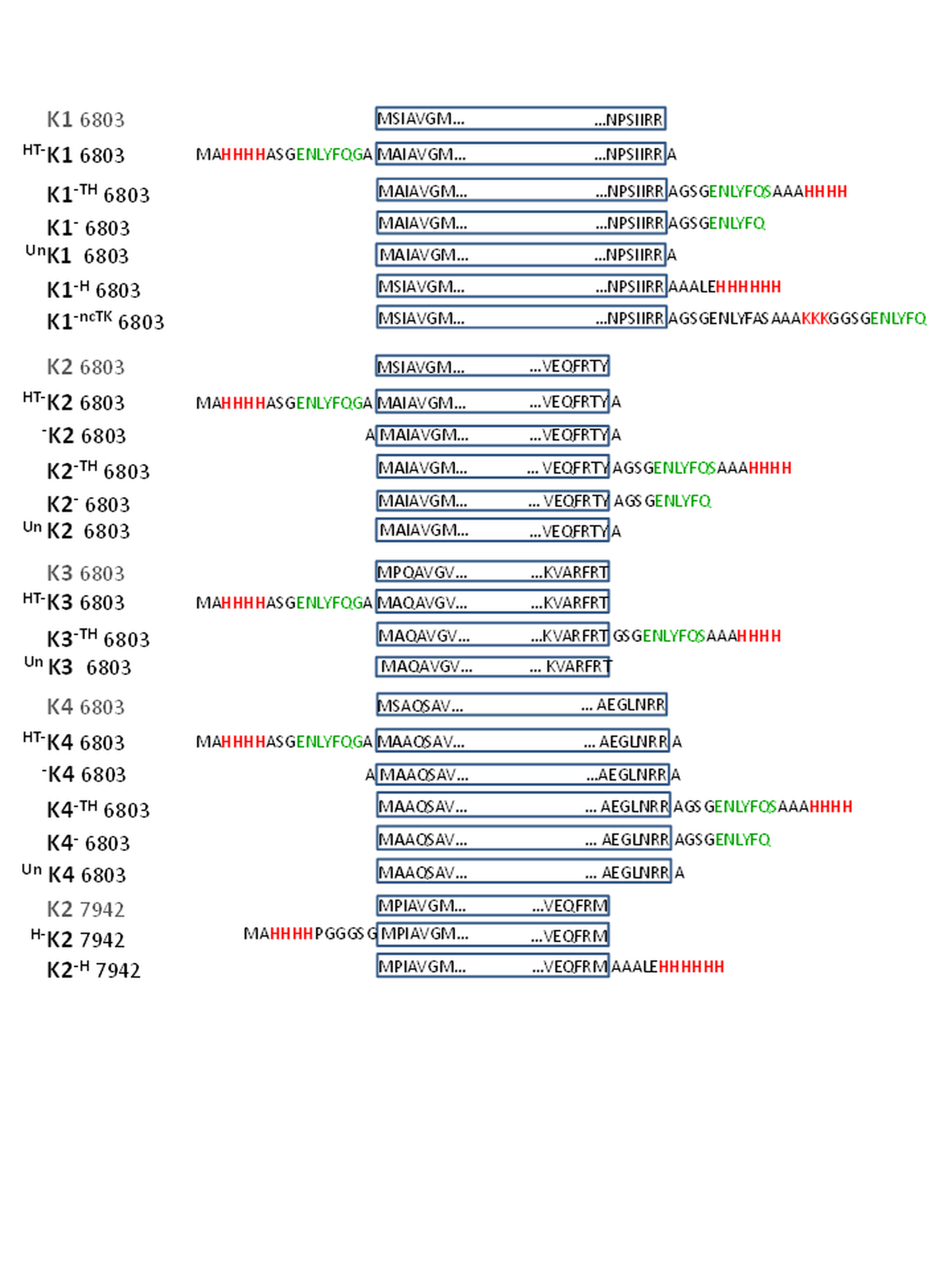

Supplement: S1 Fig — Boxes are to schematize emplacement of wild-type protein sequences, which are indicated on top for each isoform. Only starting and ending stretches are indicated within each box. N-ter or C-ter oligohistidine tags are indicated in red, in green TEV cleavable sequences. TEV-proteolyzed products are not indicated for proteins that could not be purified. (TIF) [file pone.0185109.s003.tif]

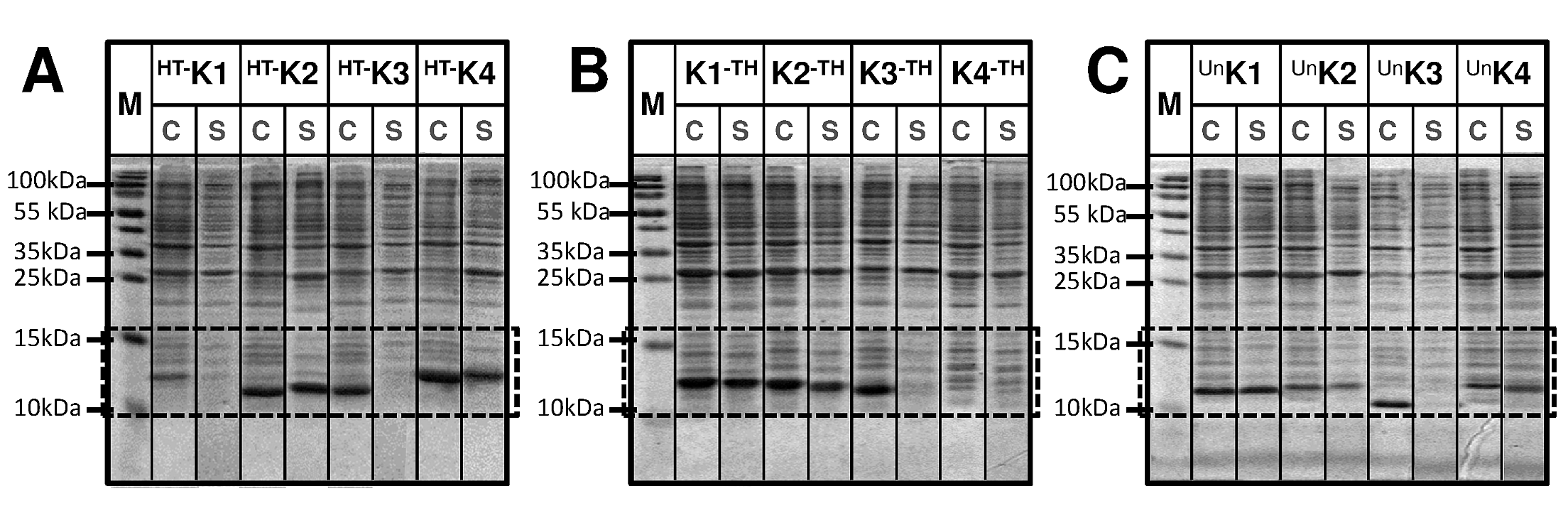

Supplement: S2 Fig — Coomassie-stained SDS-PAGE showing the presence or absence of bands corresponding to over-expressed proteins in total cell content (C) or fractions remaining soluble after lysis and centrifugation (S). Panel A: N-ter His4-tagged CcmK isoforms (HT-K); B: C-ter His4-tagged CcmK isoforms (K-TH,, B); C: untagged proteins (UnK). Proteins of interest have theoretical MW comprised between 12.8 and 14.1 kDa (region indicated with dashed box). Amounts equivalent to approximately 4 μL at OD280nm = 5 were loaded in each lane. (TIF) [file pone.0185109.s004.tif]

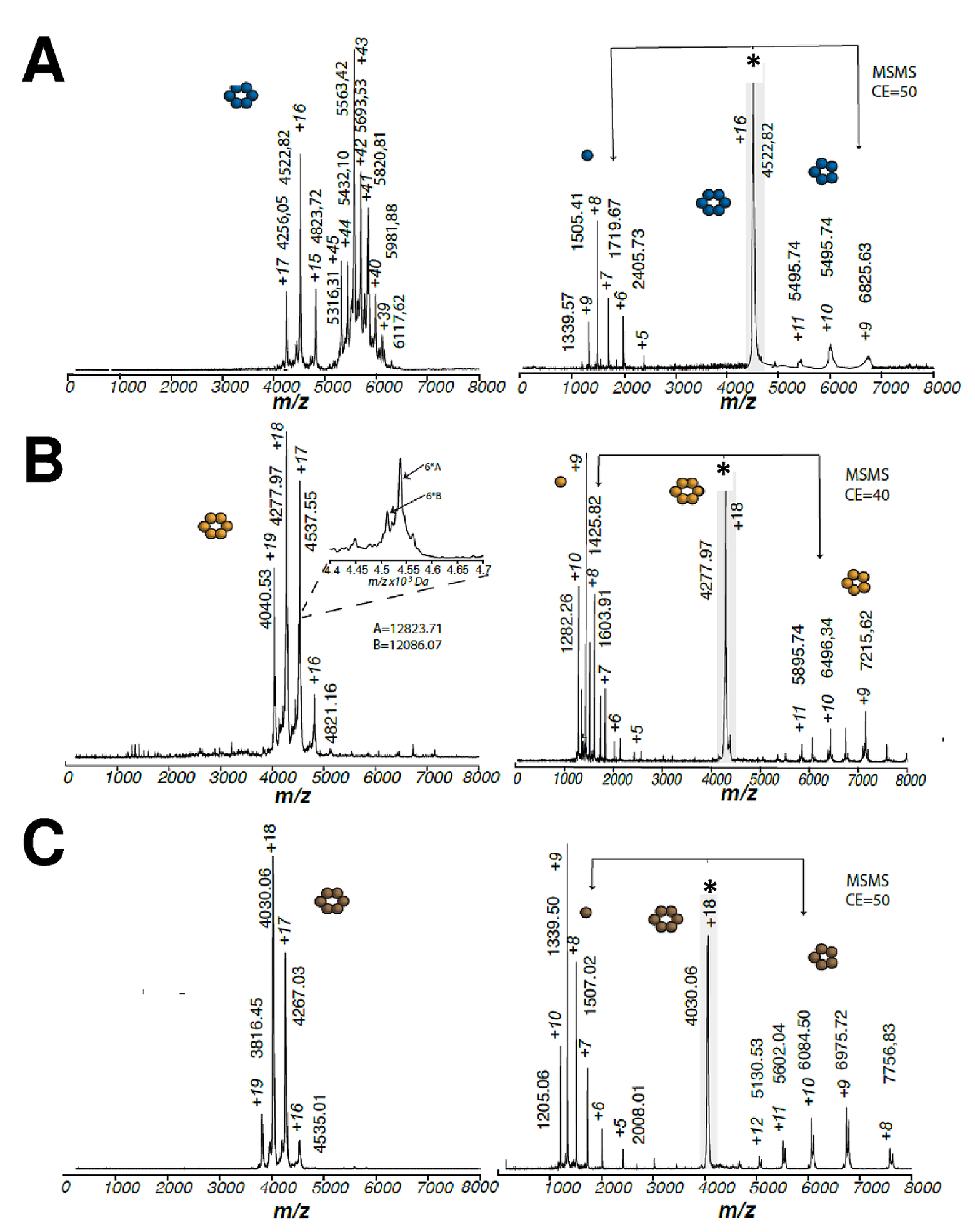

Supplement: S3 Fig — Positive-ion mode native ESI-MS spectra from CcmK isoforms: A, Syn6803 K2-; B, Syn6803 K4-; C, Syn7942 K2-H. For panels A and B, tags were removed by TEV protease treatments prior to spraying. Data support the occurrence of hexamers in solution. In addition, potential assembling intermediates with higher oligomerization state (see main text) were noticed in experiments with K2- 6803 isoforms (panel A). Species of similar, but not identical MW are detected for K4-TH (B), pointing to sample proteolytic degradation (portion enlarged in the inset, for clarity). Right panels present collisional activation data collected on selected hexamer precursor ions (asterisk). An asymmetric charge partitioning is noticed, hexamers dissociating into highly-charged monomer and pentamer species carrying the remaining charge. Species m/z values and charges are indicated above most intense peaks. Molecular weights of neutral species obtained by convolution of these data are compiled in S1 Table. (TIF) [file pone.0185109.s005.tif]

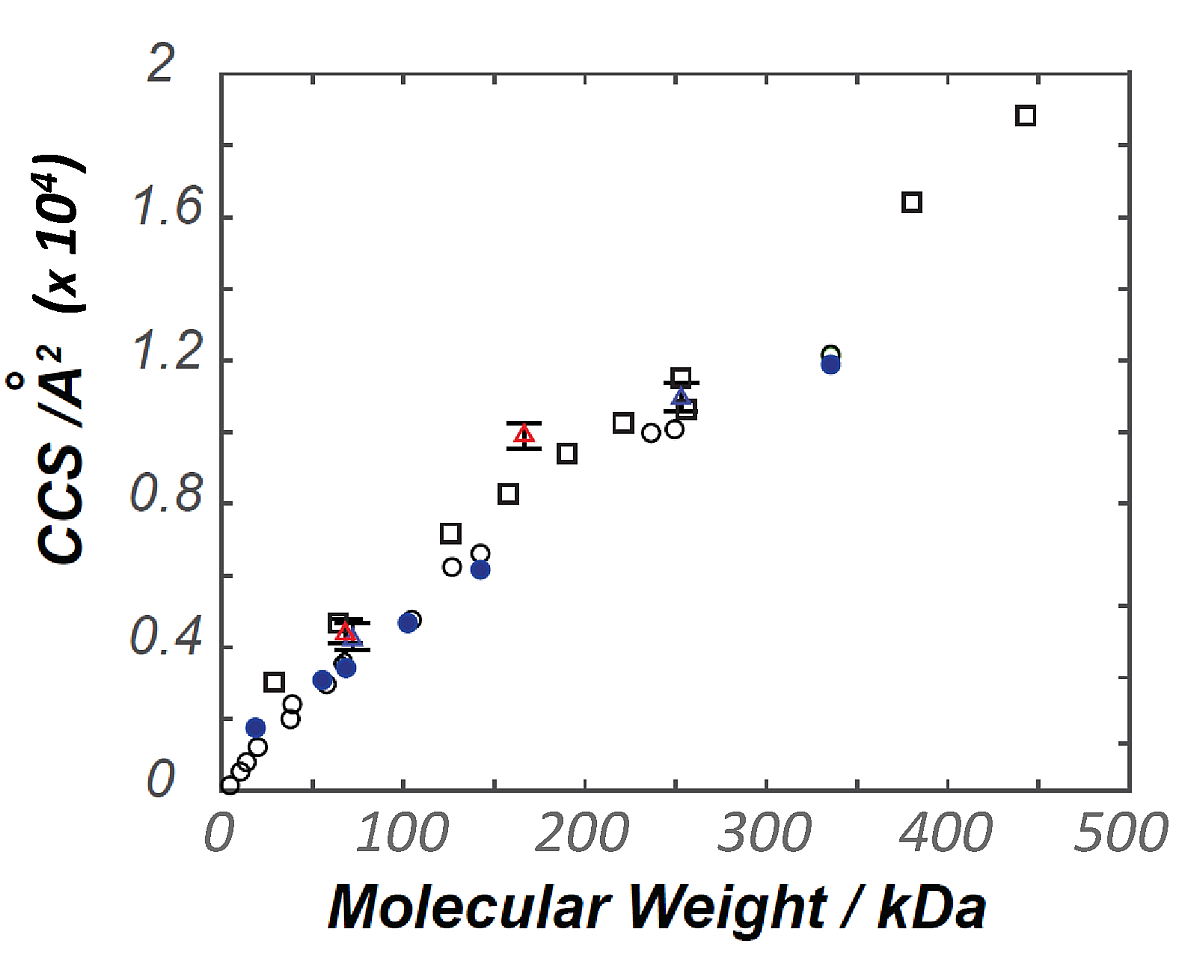

Supplement: S4 Fig — Collisional cross sections (CCS) were determined for selected species detected in Fig 1B with TEV-treated -K2 (red triangles) or in S3B Fig for K2- (blue triangles). CCS data for hexameric species (approx. MW 70 kDa) were measured on species with m/z 3799 for -K2 and m/z 4522 for K2-, whereas CCS values for higher MW species were estimated from peaks at m/z 4999 for -K2 and m/z 5563 for K2-. Experimental Ω values reported before for sheet-like partially disassembled viruses (empty squares) and globular proteins (open circles)38, as well as globular proteins measured here (blue filled circles) are plotted for comparison. (TIF) [file pone.0185109.s006.tif]

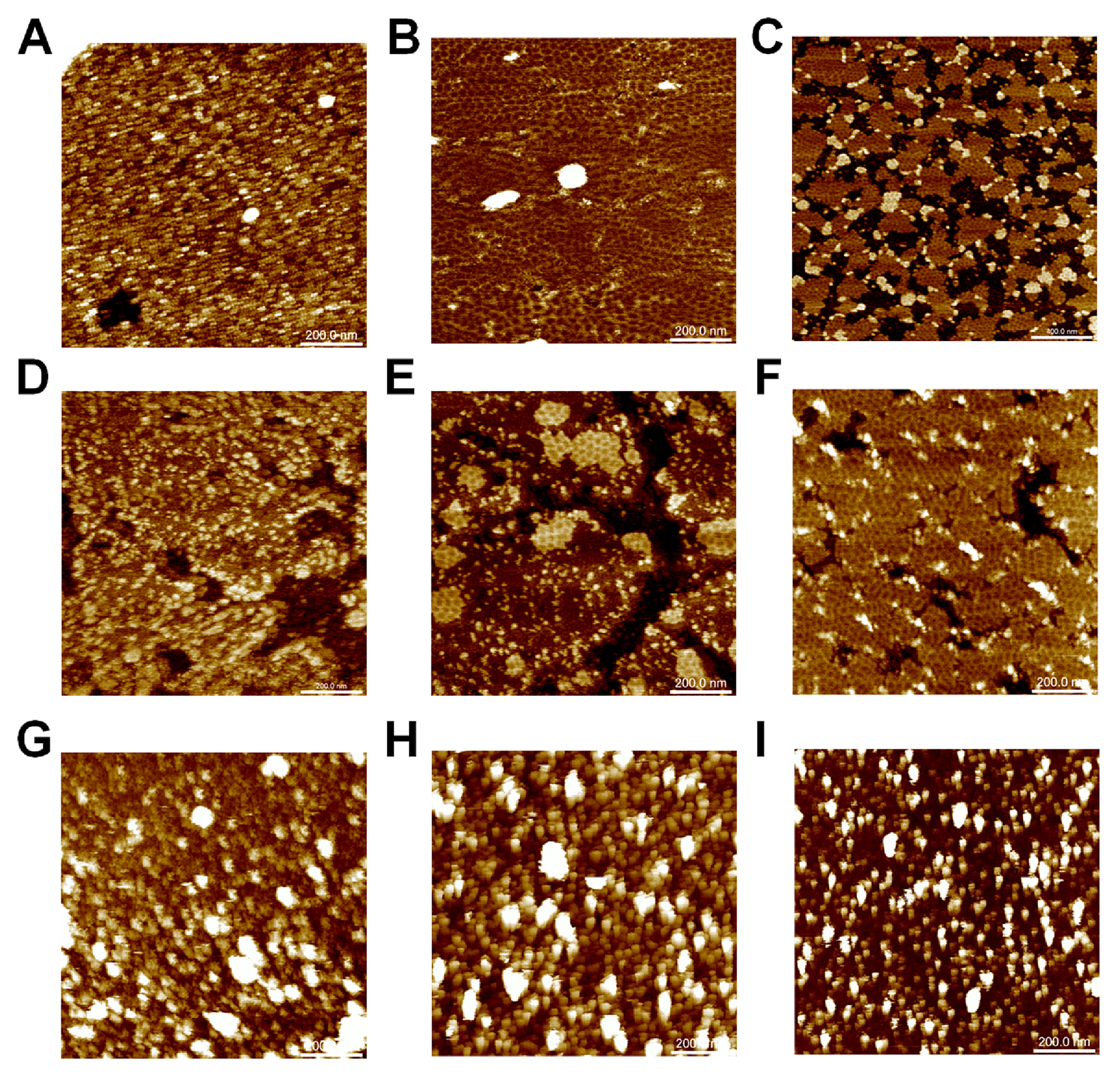

Supplement: S5 Fig — AFM images were recorded after absorption on mica of 100 ng of K1-TH 6803 conditioned in saline Tris buffer pH7.0 including next additives: A, nothing; B, 0.5mM ADP; C, 0.5 mM GtetraPi; D, 0.5 mM NaHCO3; E, 1 mM HEPES; F, 0.5 mM 3-phosphoglyceric acid; G, 0.5 mM Na2SO4; H 0.5 mM ribulose-1,5-biphosphate; I, 0.5 mM MgCl2. Images are 1 μm large, 2 μm for panel C. (TIF) [file pone.0185109.s007.tif]

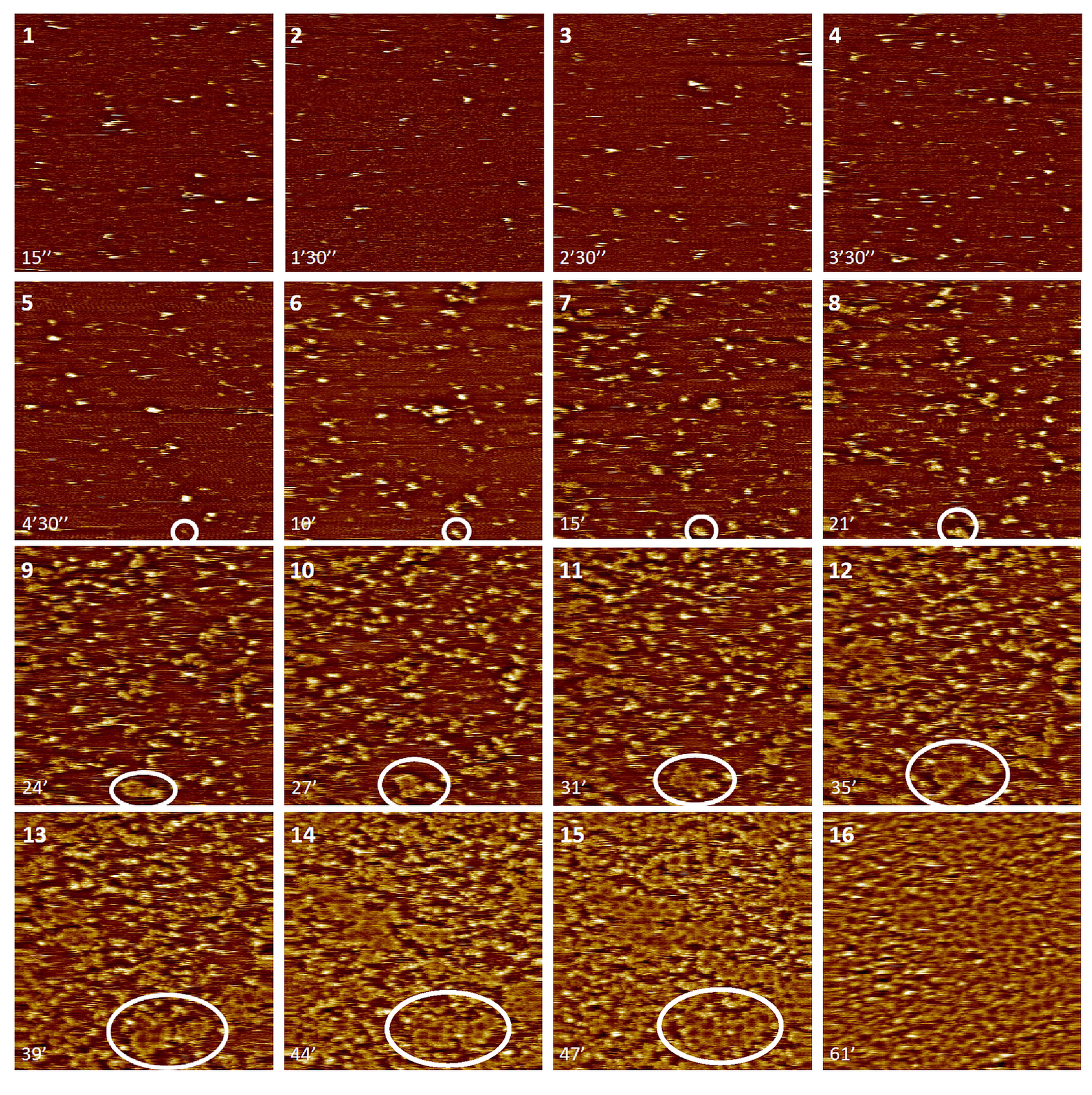

Supplement: S6 Fig — Shown are sixteen non-aligned time-lapse AFM images selected from a 1hr long HS-AFM movie recorded at 4 sec per frame (S1 Movie). White circles are depicted to indicate the emplacement of one of the earliest assembly events that lead to a curved honeycomb-like patch. Images were captured after injection of 50 μL of K1-TH 6803 (40 μg/mL) in 10 mM NaPi/300 mM NaCl at pH 6.5 once the cantilever immersed probe engaged above the mica in 50 μL of the same solution. (TIF) [file pone.0185109.s008.tif]

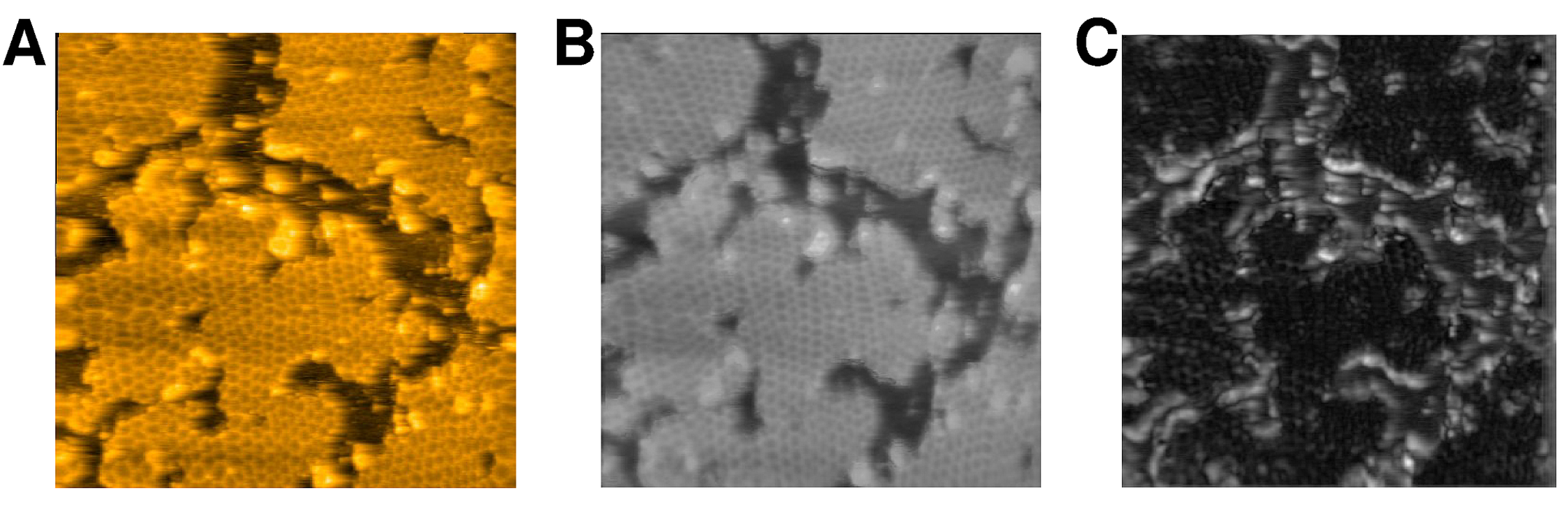

Supplement: S7 Fig — Left, single frame representative of assembled motifs monitored over 20 min at 10 sec intervals (S2 Movie). Prior to imaging, the protein was allowed to assemble for 30 min on mica in the presence of 10 mM MES, 300 mM NaCl, pH 7. Centre, average image obtained after alignment of 123 recorded frames. Right, representation of standard deviation between images, with grey scale ranging from white to black for highest difference to no change, respectively. (TIF) [file pone.0185109.s009.tif]

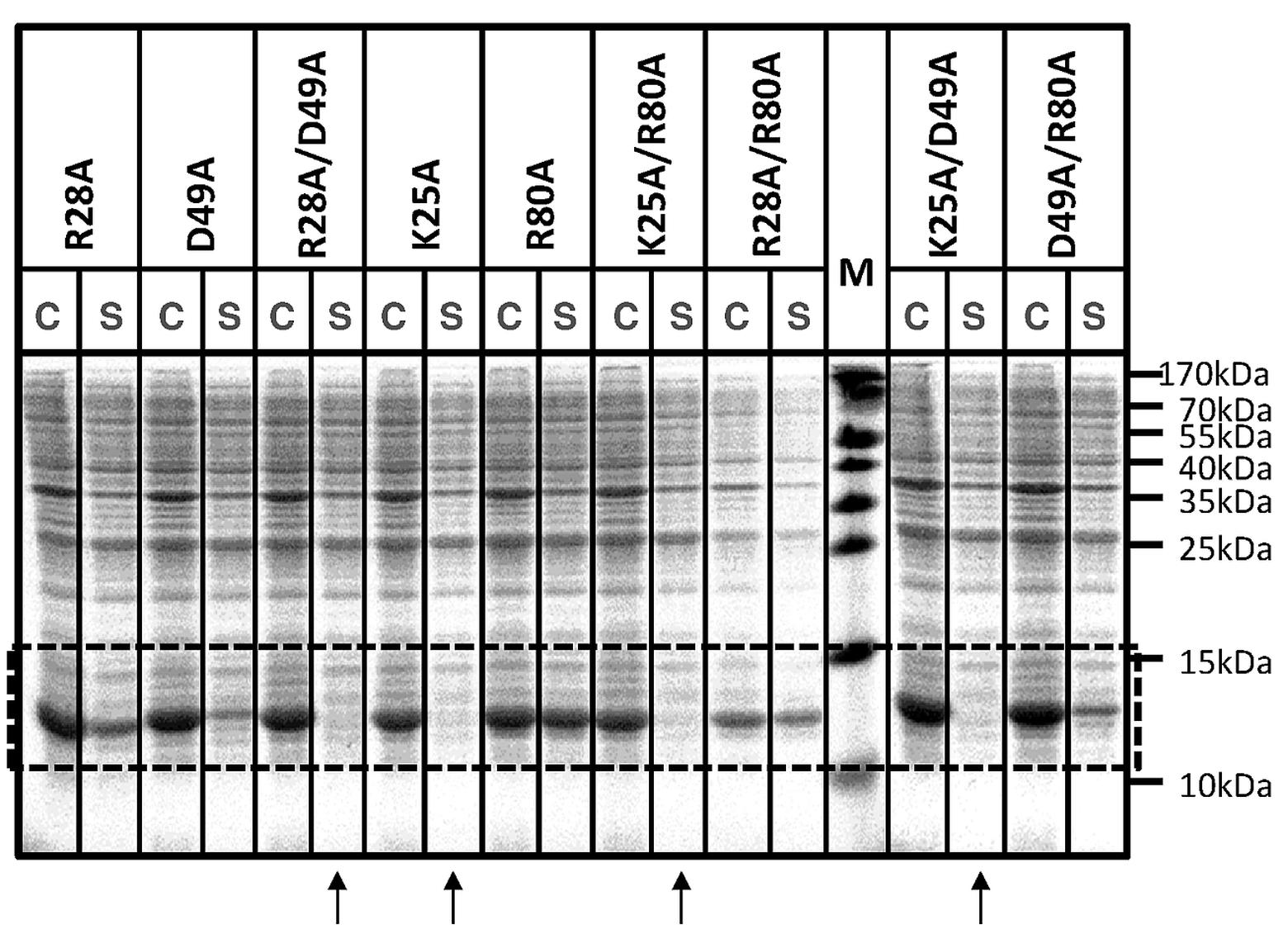

Supplement: S8 Fig — Coomassie-stained SDS-PAGE showing the presence or absence of bands corresponding to over-produced proteins (theoretical MW of 14 kDa) in total cell content (C) or fractions remaining soluble after lysis and centrifugation (S). Indicated with arrows are mutants that could not be purified. Total amount loaded in each lane is equivalent to approximately 4 μL of a protein solution at OD280nm = 5. (TIF) [file pone.0185109.s010.tif]

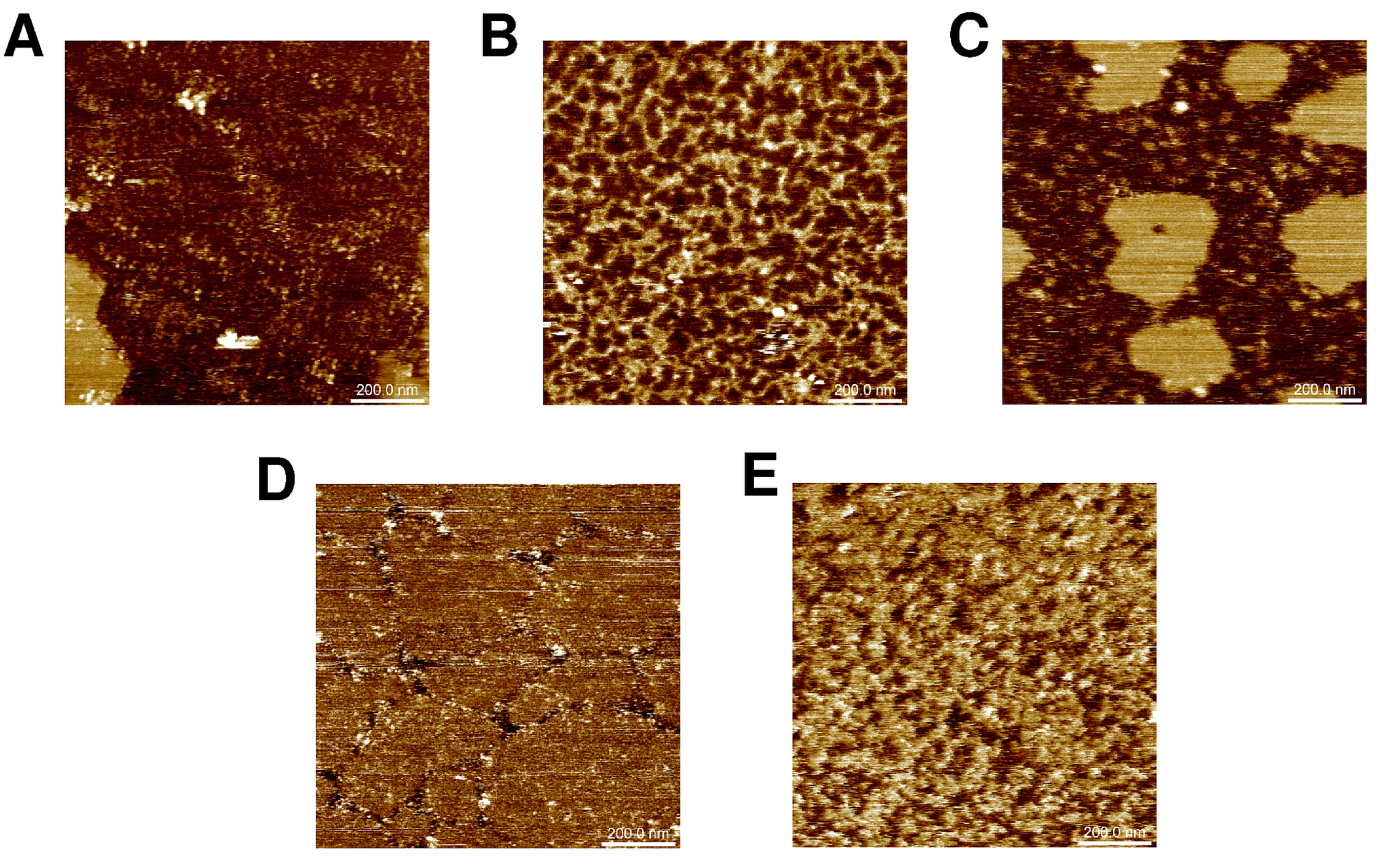

Supplement: S9 Fig — AFM images were recorded after absorption on mica of 100 ng of K1-TH 6803 indicated mutants conditioned in saline MES buffers at pH 6.0: R28A (panel A), D49A (B), R80A (C), and double mutants R28A/R80A (D) or D49A/R80A at pH 6.5 (E). Only shown one image from several performed for each mutant after deposition at pH 6.0, 6.5 and 7.0. Image sizes are 5 times those of indicated scale bars. (TIF) [file pone.0185109.s011.tif]

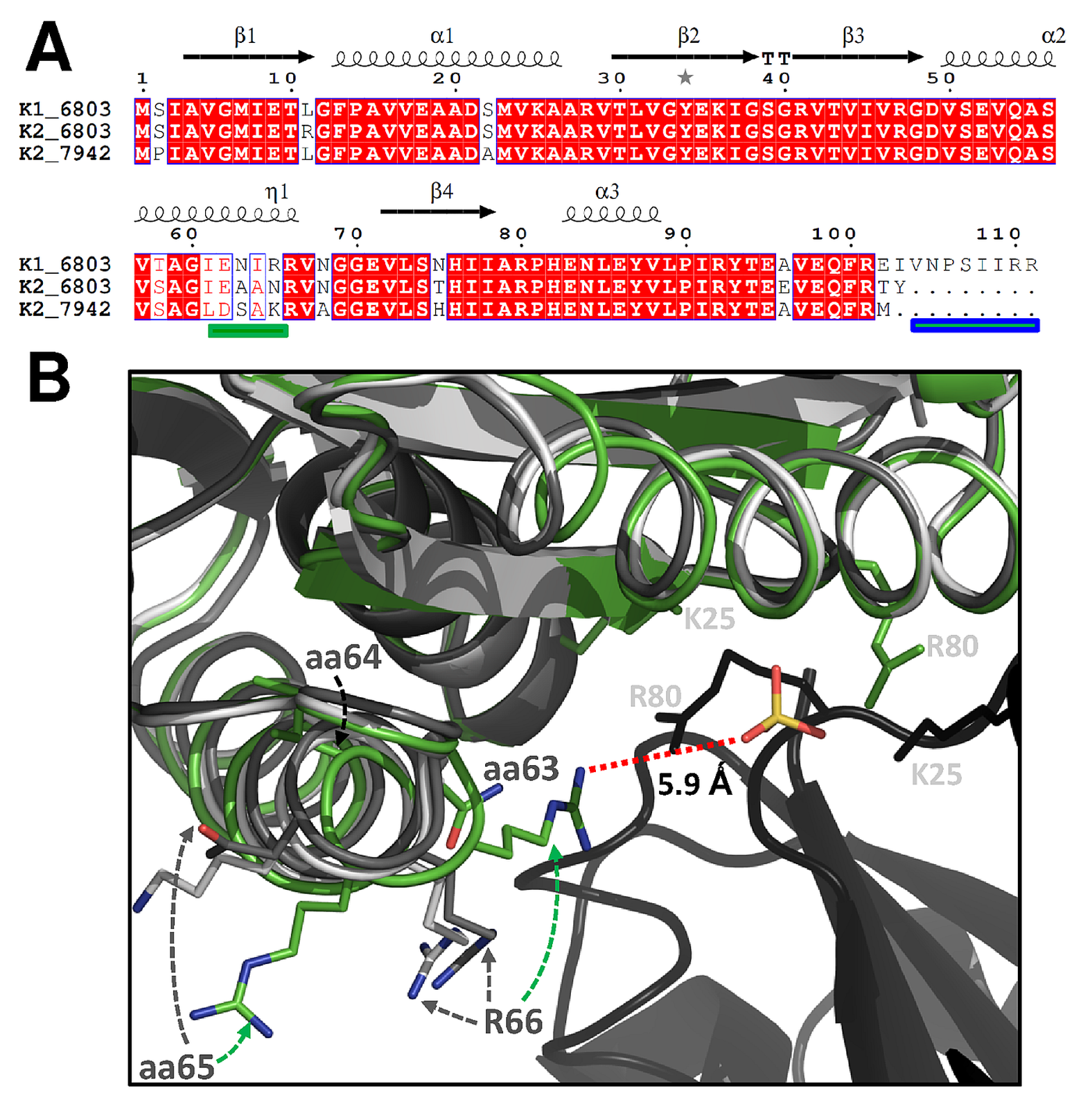

Supplement: S10 Fig — A, Sequence alignments highlighting positions differing among the three proteins. Two major regions acumulate most of differences: the C-ter side of α2 helix (green bar) and the C-ter extension (blue bar) present only in K1 6803 (also in K4 6803, not shown). Secondary structural elements were extracted from K1 6803 structure (PDB ID 3BN4) are indicated on top of the alignment. B, Structural differences observed for C-ter side in helix α2 of K1 6803 (3BN4, in green) as compared to K2 6803 (2A1B, dark grey) or K2 7942 (4OX7, light grey). Side-chain atoms of some selected residues are represented as sticks: carbon with same colors as cartoon, nitrogens in blue and oxygens red. A neighboring hexamer generated with symmetry operations on 3BN4 structure is shown on the bottom right side (black cartoon/sticks). Modeled sulfate is represented with sulfur and oxygen atoms as yellow and red sticks, respectively. (TIF) [file pone.0185109.s012.tif]

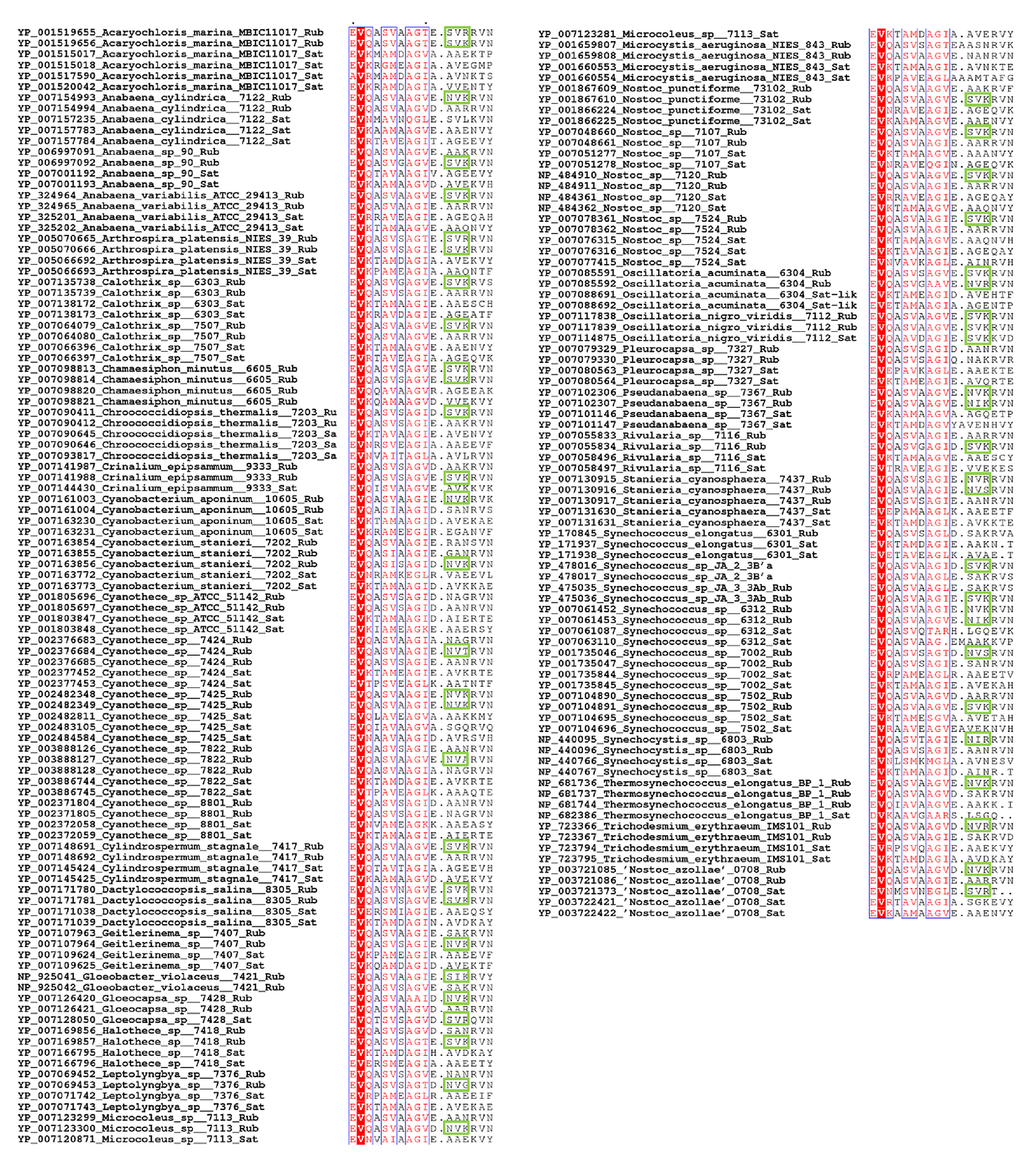

Supplement: S11 Fig — Only shown sequence stretch around residues corresponding to amino acids 63–66 of CccmK1 from Syn Sp. PCC6803. Sequence names: NCBI entry code_species name_Rub/Sat, where Rub or Sat indicate whether the corresponding ORF lies at the same loci as RuBisCO subunits or in a satellite loci [as defined in reference [1]]. Green frames highlight sequences that presumably might lead to similar structural consequences as NI 63–64 residues of Syn6803 CcmK1. (TIF) [file pone.0185109.s013.tif]

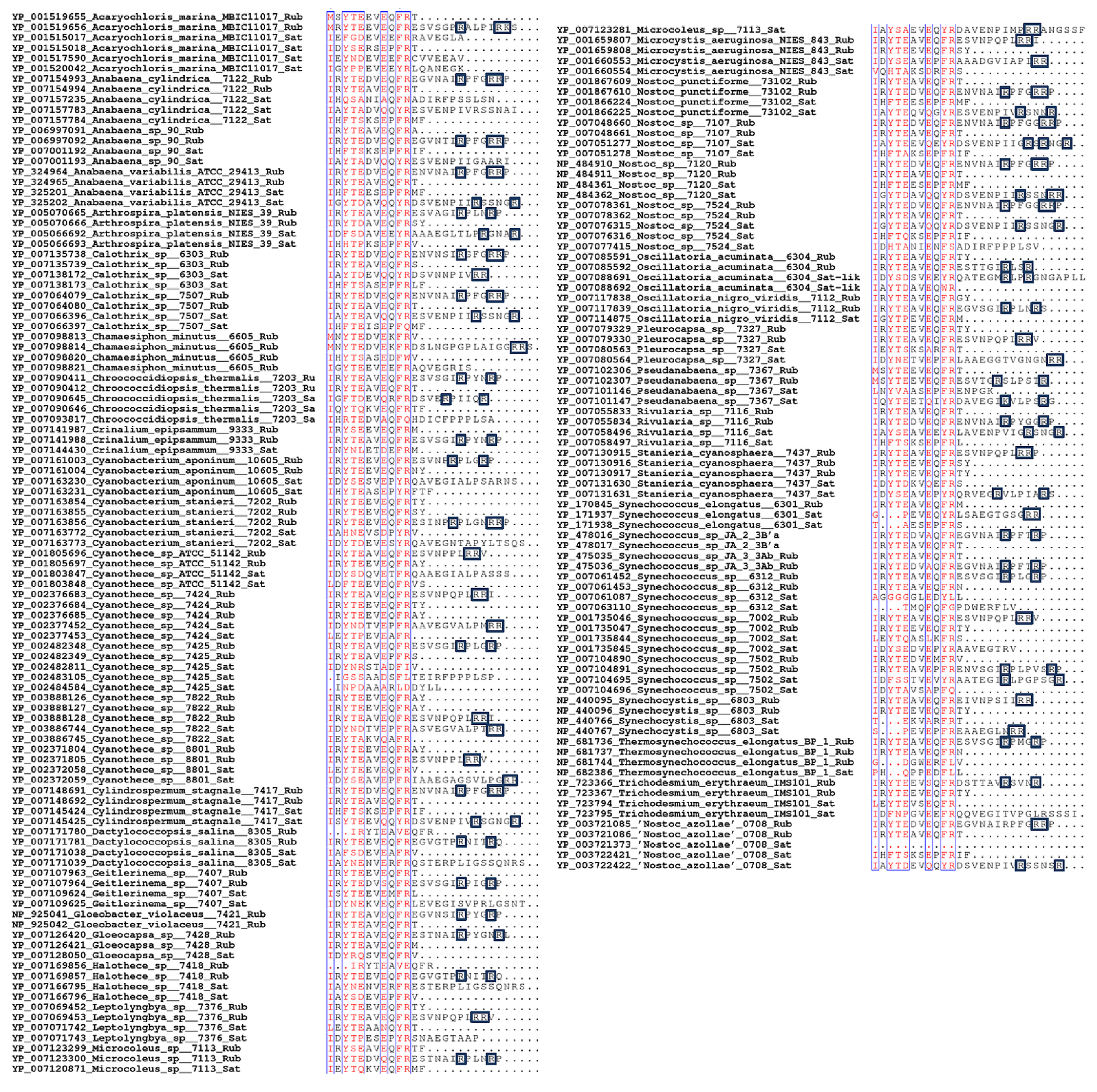

Supplement: S12 Fig — Only shown sequence stretch around residues corresponding to the C-terminal extension. Other details as for S11 Fig. (TIF) [file pone.0185109.s014.tif]
